# Machine learning for autism spectrum disorder diagnosis using structural magnetic resonance imaging: Promising but challenging

**DOI:** 10.3389/fninf.2022.949926

**Published:** 2022-09-28

**Authors:** Reem Ahmed Bahathiq, Haneen Banjar, Ahmed K. Bamaga, Salma Kammoun Jarraya

**Affiliations:** ^1^Department of Computer Science, Faculty of Computing and Information Technology, King Abdulaziz University, Jeddah, Saudi Arabia; ^2^Center of Artificial Intelligence in Precision Medicines, King Abdulaziz University, Jeddah, Saudi Arabia; ^3^Neuromuscular Medicine Unit, Department of Pediatric, Faculty of Medicine and King Abdulaziz University Hospital, King Abdulaziz University, Jeddah, Saudi Arabia

**Keywords:** autism spectrum disorder (ASD), biomarkers, structural magnetic resonance imaging (sMRI), machine learning, deep learning

## Abstract

Autism spectrum disorder (ASD) is a complex neurodevelopmental disorder that affects approximately 1% of the population and causes significant burdens. ASD’s pathogenesis remains elusive; hence, diagnosis is based on a constellation of behaviors. Structural magnetic resonance imaging (sMRI) studies have shown several abnormalities in volumetric and geometric features of the autistic brain. However, inconsistent findings prevented most contributions from being translated into clinical practice. Establishing reliable biomarkers for ASD using sMRI is crucial for the correct diagnosis and treatment. In recent years, machine learning (ML) and specifically deep learning (DL) have quickly extended to almost every sector, notably in disease diagnosis. Thus, this has led to a shift and improvement in ASD diagnostic methods, fulfilling most clinical diagnostic requirements. However, ASD discovery remains difficult. This review examines the ML-based ASD diagnosis literature over the past 5 years. A literature-based taxonomy of the research landscape has been mapped, and the major aspects of this topic have been covered. First, we provide an overview of ML’s general classification pipeline and the features of sMRI. Next, representative studies are highlighted and discussed in detail with respect to methods, and biomarkers. Finally, we highlight many common challenges and make recommendations for future directions. In short, the limited sample size was the main obstacle; Thus, comprehensive data sets and rigorous methods are necessary to check the generalizability of the results. ML technologies are expected to advance significantly in the coming years, contributing to the diagnosis of ASD and helping clinicians soon.

## Introduction

Autism spectrum disorder (ASD) is a neurodevelopmental disorder characterized by early deficits in social interactions and communication and restricted and repetitive activities and interests (American Psychiatric Association, 2013). As its name suggests, rather than a single condition, ASD includes a wide range of symptoms (Tanu and Kakkar, 2019) that reflect an overarching diagnostic category that, before the fifth edition of the Diagnostic and Statistical Manual of Mental Disorders (DSM-5), was comprised of multiple separate disorders, such as Autistic disorder, Asperger’s syndrome, and other pervasive developmental disorders (American Psychiatric Association, 2013). Language difficulties, epilepsy, and other problems may come with ASD (Yasuhara, 2010; Sivapalan and Aitchison, 2014). The prevalence of ASD in the United States has grown from 1 in 110 to 1 in 69 over 4 years (from 2008 to 2012) (Lee and Meadan, 2021). Approximately 1% of the world’s population was diagnosed with ASD, and its prevalence among males is four times higher than among females (World Health Organization, 2013; Gao K. et al., 2021). ASD’s etiology is still elusive, but genetics and the environment may play a role (Chaddad et al., 2017).

Early diagnosis and effective intervention improve the quality of life for autistic individuals (Pagnozzi et al., 2018). The gold standard for diagnosing most mental disorders, including ASD, is observation and visual analysis of behavior, such as interviews (Zhang L. et al., 2020; Jarraya et al., 2021). These instruments, however, have limits. A child’s apparent inability to cope with their environment is usually diagnosed at 3 years old (Wang et al., 2018). Interpretive coding of children’s observations is time-consuming (Eslami et al., 2021). Comorbidities and other disorders that share prominent features with ASD may hinder diagnostic evaluation (Gargaro et al., 2011). Clinical training, tools, and cultural context may influence a clinician’s subjective observations (Eslami et al., 2021). These limitations necessitate more optimal diagnosis methods, such as biomarker-based diagnosis.

In the 1990s, MRI techniques such as structural MRI (sMRI) and functional MRI (fMRI) evolved significantly (Libero et al., 2015; Eslami et al., 2021). Each modality gives unique information about the brain (Ozonoff et al., 2011; Wang et al., 2018). sMRI can dissect brain structures in different images, such as T1 and T2 weighted, and T2-weighted Fluid Attenuated Inversion Recovery (FLAIR) (Mostapha, 2020; Eslami et al., 2021). sMRI also tracks brain growth over time in longitudinal studies, showing early life risk factors (Li et al., 2019a). fMRI tracks changes in blood flow to brain regions that stimulate neurons (Mostapha, 2020). The two main forms of fMRI are rs-fMRI and task fMRI (Xu et al., 2021).

Numerous studies have explored brain abnormalities in the cerebellum (Sivapalan and Aitchison, 2014), gray matter (GM) volumes (Rojas et al., 2006), and brain functional connectivity (FC) (Nomi and Uddin, 2015), and others using statistical methods (Polsek et al., 2011). However, the interdependency between diverse brain regions has been neglected (Libero et al., 2015; Eslami et al., 2021). In addition, group differences are not individual differences, so results from research cannot be directly translated into clinical practice (Rojas et al., 2006; Xu et al., 2021). Machine learning (ML) models and deep learning (DL) techniques have recently become attractive to be applied in the diagnosis of diseases like Parkinson’s (Manzanera et al., 2019) and epilepsy (Abbasi and Goldenholz, 2019). Conventional ML methods facilitate the exploration of complex abnormal imaging patterns and consider the relationships between different brain regions (Xu et al., 2021). Thus, it can greatly enhance the role of statistical methods. Computer-aided diagnostic (CAD) systems are also a low-cost method that reduces healthcare expenditure compared with other methods. They’re simple enough that even computer scientists with no prior training in psychiatry can analyze data and extract insights (Manzanera et al., 2019; Eslami et al., 2021). In ML, the key features are usually extracted manually and then tell the algorithm how to make a prediction or classification by consuming more information. For problems with complex nonlinear relationships, the DL algorithm is better suited because it learns features automatically and its performance is superior in image analysis fields, such as object detection and image classification (Zhang et al., 2021). However, the diagnosis of ASD remains a formidable challenge, as studies based on ML have shown different results that may reflect the diversity of behavioral symptoms of the disorder and its proposed etiology, often linked to the brain (Sivapalan and Aitchison, 2014).

Several publications (Zhang L. et al., 2020; Zhang et al., 2021; Quaak et al., 2021) have reviewed the classification of ASD using only ML or DL algorithms. Some representative examples of previous reviews are listed in Table 1.

**TABLE 1:** ASD application review papers based on ML and DL methods with sMRI data.

References	No. papers reviewed	Years covered	Methods covered	Diseases/disorders
Arbabshirani et al. (2017)	200	2001–2015	ML on sMRI, fMRI, Diffusion MRI, and positron emission tomography	Schizophrenia, mild cognitive impairment, Alzheimer’s disease, depressive disorders, ASD, and ADHD.
Pagnozzi et al. (2018)	123	2007–2018	ML and non-ML on sMRI	ASD
Tanu and Kakkar (2019)	48	2007–2018	ML and DL: Invasive and non-invasive diagnosis approaches	ASD
Nogay and Adeli (2020)	46	2009–2020	ML and DL on sMRI, fMRI	ASD
Khodatars et al. (2021)	82	2016–2020	DL on different autism diagnosis and rehabilitation approaches	ASD
Zhang et al. (2021)	74	2011–2021	ML and DL on different neuroimaging data	Alzheimer’s disease, Parkinson’s, major depressive disorder, schizophrenia, ADHD, and ASD.
Eslami et al. (2021)	75	2010–2020	ML and DL on different neuroimaging data	ADHD, and ASD.

However, these publications often cover many human tissues or diseases (Quaak et al., 2021). Thus, this survey focuses on ASD and brain imaging only. We should note that besides MRI techniques, other forms of brain data, such as electroencephalography (Ibrahim et al., 2018), and computed tomography (Hashimoto et al., 2000), are utilized to investigate ASD. However, MRI is the safest method due to its low radiation (Sivapalan and Aitchison, 2014). Given the high anatomical accuracy of sMRI and its availability in clinics, it is considered the most feasible method available to contribute to clinical practice (Kim and Na, 2018). In addition, capturing sMRI images requires less time and effort from patients and clinicians than other MRI methods such as fMRI (Kim and Na, 2018). We collected research on the conventional ML and/or DL directions for classifying ASDs. Unlike most published papers, ours discusses current research findings from two perspectives: medical (related to sMRI-based biomarkers associated with ASD) and technical (related to learning models, accuracy, and methods used to extract and analyze data). 45 research papers were reviewed to assess ML and DL methods for classifying ASD using sMRI. Articles were collected from abstract searches in the Web of Sciences, Scopus, and PubMed databases between January 2017 and January 2022 using the following formula: autism* AND (imaging OR MRI OR sMRI) AND (machine Learning OR deep learning) AND (classif* OR predict* OR diagnosi*).

**FIGURE 1:**
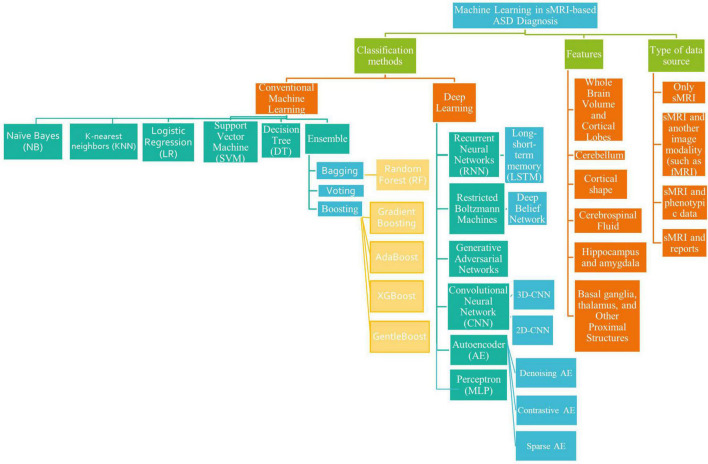
A literature-based taxonomy for ML-based ASD classification.

The survey taxonomy describes the methods, scan techniques, and features that are based on brain classification for ASD diagnosis (see Figure 1). This study looks at the age and number of subjects, features/biomarkers, types of scan modalities, datasets used, preprocessing tools, classification algorithms, and evaluation measures.

After the introduction, there are six sections in this article. Section 2 discusses the sMRI features and methods of extracting them. In Section 3, the general pipeline for ML and algorithms common in ASD research is described. In Section 4, ML/DL’s recent applications for diagnosing ASD using sMRI are presented, along with a description of the most consistent discriminatory biomarkers for diagnosing ASD across studies. Before concluding, we will evaluate the present research’s limits and discuss future directions that we believe will help researchers decide which studies to conduct. The review also provides a tabular summary of all articles, allowing readers to evaluate the area swiftly.

In summary, the purpose of this review is (a) to demonstrate ML/DL progress in brain ASD classification and (b) to identify open research challenges for developing effective ML/DL classification methods for the autistic brain.

## Structural magnetic reasoning imaging and features extraction

Including MRI, all medical imaging techniques are diagnostic in themselves. It generates non-invasive visual representations of the body’s interior that are utilized to extract insights for clinical evaluations and describe pathological processes (Zhang L. et al., 2020).

**FIGURE 2:**
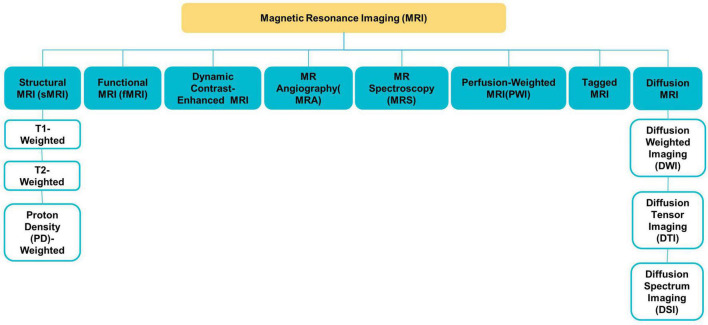
Different MRI modalities.

Various MRI modalities, such as sMRI, fMRI, and diffusion tensor imaging (DTI) (see Figure 2), have been employed in studies to capture the effect of ASD on the brain from a range of perspectives (Ahmad et al., 2014).

sMRI is commonly used to examine brain morphology because of its high contrast sensitivity, spatial resolution, and the fact that it does not need exposure to ionizing radiation; this is especially significant for children and adolescents (Ali et al., 2022). sMRI delivers various sequences of brain tissue (e.g., T1, T2, and FLAIR) created by altering excitation and repetition durations to view multiple brain regions (Eslami et al., 2021).

With the explosion in data in medical imaging of various types, medical image analysis and the extraction of clinically relevant information have become a major challenge. AI technologies are needed to enhance health care outcomes by boosting sophisticated analytical skills. Medical imaging research on CAD is expanding quickly. Because items such as organs may not be represented accurately by a simple equation; thus, medical pattern recognition requires “learning from examples” (Suzuki, 2013). One of the most popular uses of ML is the classification of objects such as brains into certain classes (e.g., healthy or autistic) based on input features (e.g., GM volume).

In CAD systems, the sMRI image undergoes steps like acquisition, image enhancement, feature extraction, the region of interest (ROI) definition, result interpretation, etc. Feature extraction conducts scientific, mathematical, and statistical operations or algorithms to discover quantifiable features/biomarkers from an sMRI image which can be used as inputs to ML models to detect brain disorders. Morphometric features and morphological networks are the two main types of features that can be extracted from sMRI.

This section discusses the most used methods for defining features from sMRI data.

### Morphometric features

Morphometric features include two main types, geometric and volumetric features, which can be employed for the MRI-based diagnosis of ASD. Geometric features are two-dimensional surface features associated with the cerebral cortex, such as curvature, surface area, and thickness (Ali et al., 2022). While volumetric features usually refer to the size of the subcortical structures [e.g., white matter (WM) volume] (Ecker et al., 2010). Some tools, such as FreeSurfer, and Statistical Parametric Mapping (SPM), can easily extract morphometric features (Shen et al., 2017).

### Morphological networks

This method connects morphological data from various brain regions (Eslami et al., 2021).

Depending on the spatial scale, features can be produced in one of three ways: voxel-based, region-based, or network-based (Xu et al., 2021).

Researchers can use pre-defined regions and extract data specifically from voxels within those regions to find specific findings in brain scans. This is called an ROI-based analysis (Chen et al., 2011). Experts manually or semi-manually identify brain regions, which takes a long time. This type of research is also limited by the number of brain regions that can be examined. By increasing the number of voxels in a target ROI, the statistical power increases (Chen et al., 2011). ROI detection algorithms fall into four categories: (1) based on changes in voxel values, like edge detection algorithms; (2) based on human-computer interaction. (3) those that use human visual characteristics, such as color detection algorithms; (4) DL-dependent, like Recurrent Attention Model (RAM) and Class Activation Mapping (CAM) (Ke and Yang, 2020).

In contrast, voxel-based approaches can detect statistically significant tissue density differences between the two groups (Seyedi et al., 2020). It is more appropriate given the lack of consensus on which brain areas are important in ASD (Eslami et al., 2021). Voxel-wise techniques include voxel-based morphometry (VBM), surface-based morphometry (SBM), and tensor-based morphometry (TBM) (Chen et al., 2011; Chen T. et al., 2020). VBM’s main characteristics are the density and volume of GM, WM, and cerebrospinal fluid (CSF) (Chen et al., 2011). TBM, unlike VBM, does not compute volume information for various tissue types separately (Chen T. et al., 2020). On the other hand, SBM research focuses on cortical topographic measurements like thickness, curvature, and area (Chen et al., 2011). The SBM method excludes ASD-linked subcortical regions like the basal ganglia (Chen T. et al., 2020). Neurological disease can damage multiple brain regions, making voxel-based or region-based approaches ineffective (Islam, 2019). Network-based methods are used to extract global features like voxel or ROI interaction patterns (Eslami et al., 2021).

## General machine learning pipeline and common algorithms for classification of autism spectrum disorder

**FIGURE 3:**
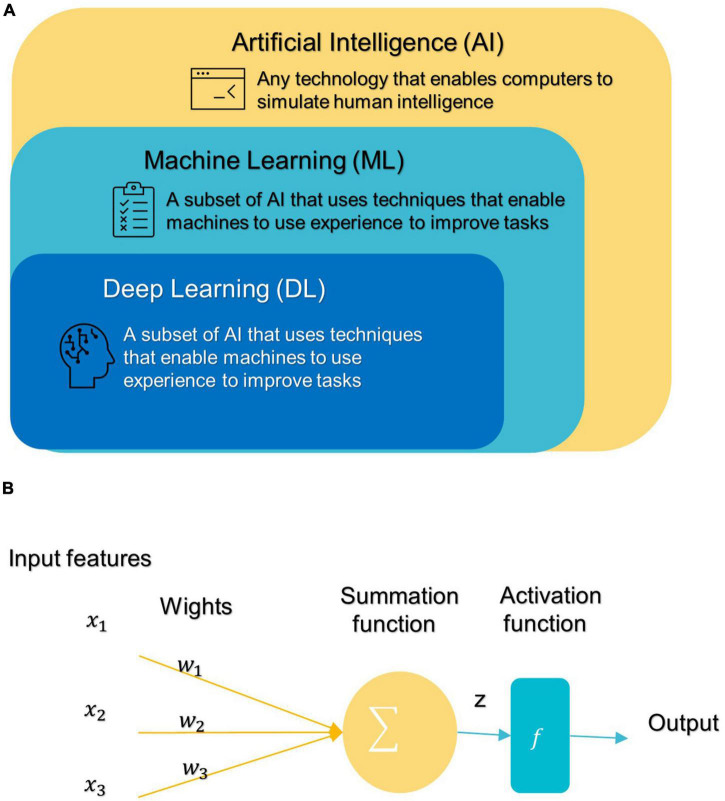
**(A)** Branches of Artificial Intelligence Science. **(B)** An artificial neuron’s architecture. Each input × is associated with a weight w. The sum of all weighted inputs is passed onto an activation function f that leads to an output.

In 1959, Samuel coined the term “machine learning” (ML), which is a subfield of artificial intelligence (AI) that allows machines to learn from data without being explicitly programmed (see Figure 3A; Samuel, 2000; El Naqa and Murphy, 2022). ML has three broad categories: supervised, unsupervised, and semi-supervised learning algorithms (Eslami et al., 2021). Deep learning (DL) is a subset of ML that is based on artificial neural networks inspired by the way human neurons communicate (Eslami et al., 2021).

Most conventional ML algorithms required human intervention to extrapolate specific data features and patterns before consuming them to learn from Eslami et al. (2021). Handcrafted feature extraction is an expensive procedure (Nogay and Adeli, 2020). DL can automatically detect and extract representations (features) with strong discriminatory power from input data (Liu et al., 2020).

**FIGURE 4:**
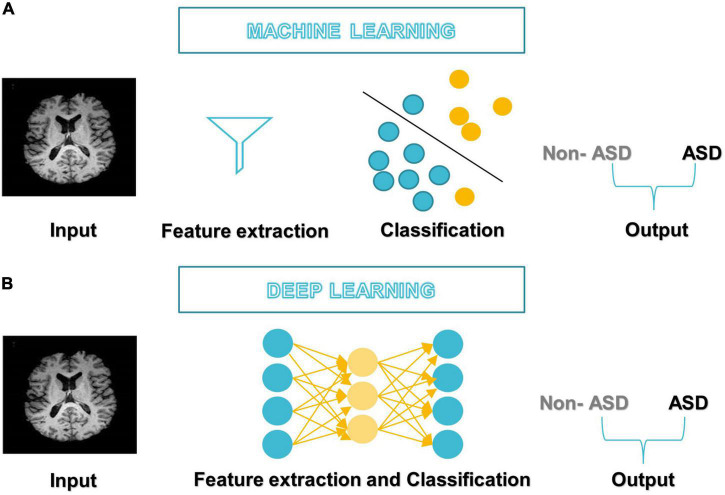
Differences between **(A)** ML-based studies workflow and **(B)** DL-based studies workflow.

After DL’s success in the ImageNet challenge in 2012, it only took 5 years for the first DL algorithm for medical imaging (Krizhevsky et al., 2012). A deep neural network (DNN) (Misman et al., 2019) has one or more hidden layers between the input and output layers. Each layer is made up of layers of nodes known as artificial neurons (see Figure 3B). Each layer’s representation is transformed into the most abstract and composite layer. The purpose of hidden layers is to automatically collect valuable features from input and apply them in the classification stage (Liu et al., 2020). Figure 4 represents the difference between ML and DL.

Therefore, the application of ML models that also include DL to diagnose disorders has increased rapidly in recent years. So, in the next section, we focus on describing ML models and the general framework for their application in ASD diagnostic studies to make them accessible to neuroscientists.

### A general machine learning based framework for classification of autism spectrum disorder

**FIGURE 5:**
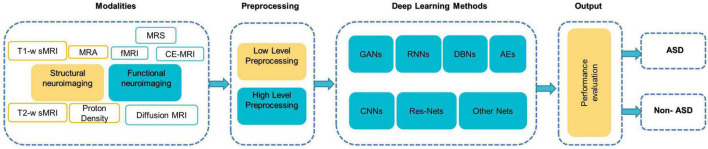
The components of typical DL-based methods for diagnosing ASD (Khodatars et al., 2021).

Figure 5 illustrates a generic pipeline for establishing an ML-based ASD diagnosis. ASD classification may be broken down into four components: (a) data collection and preprocessing; (b) feature extraction and selection/reduction; (c) model training; and (d) model testing and performance evaluation.

#### Data acquisition and preprocessing

The first step in building a good classification framework is always to obtain the right data that represents the entire field of interest, is suitable for the learning objective, and is consistent, complete, and adequate.

Preprocessing is then mainly used to enhance the visual impression of an image. Due to the complex structure of neuroimaging data, failure to preprocess them might have a negative impact on the final diagnosis (Wujek et al., 2016; Khodatars et al., 2021). There are two layers to the preprocessing of neuroimaging data: low-level processing and high-level processing. Low-level processing steps such as brain extraction, normalization, spatial smoothing, and atlas registration [e.g., automated anatomical labeling (AAL), Harvard Oxford Atlas (HO)] are frequently repeated across studies and are typically performed with pre-built toolboxes [e.g., FreeSurfer (Fischl, 2012), FSL (Jenkinson et al., 2012), iBET (Dai et al., 2013), and SPM (Khodatars et al., 2021) to minimize processing time and improve a study’s reproducibility (Khodatars et al., 2021)]. High-level processing [e.g., data augmentation (DA) (Eslami et al., 2019), and sliding window (Li X. et al., 2018)] is applied to the data following typical preprocessing methods to increase the accuracy of ASD detection. It is difficult to implement and repeat complex processing steps in neuroimaging, so pipelines such as Nipype or LONI have been found that combine the power of analytical tools with the speed of data processing as well as facilitate the repetition of the same steps between different studies (Khodatars et al., 2021).

#### Feature extraction, selection/reduction

A feature is any measurable property extracted from the source dataset regarding the class. Through features engineering, neuroimaging data is transformed into trustworthy and biologically relevant features that greatly influence data separation (Xu et al., 2021). The “dimensionality curse” problem is quite common in medical imaging analysis because the sample density decreases exponentially as the number of features increases. Some of these features may be redundant or irrelevant to the prediction; removing them does not result in a significant loss of information (Xu et al., 2021). If there are too many features compared to the number of samples, then more training samples will be required; otherwise, there is a risk of model “overfitting,” which causes the model to perform well on training data but badly on unseen or new data; such models are deemed non-generalizable (Kim and Na, 2018).

The most efficient technique for avoiding the curse of dimensionality is feature selection/reduction, which reduces noise and redundant features and facilitates the understanding of neural mechanisms of diseases by preserving the most discriminant features while increasing model accuracy and generalizability (Kim and Na, 2018). There are two basic ways to feature selection: supervised and unsupervised. Supervised approaches need the training label to choose informative and discriminative feature dimensions and exclude others (e.g., exclude irrelevant variables) (Saeys et al., 2007). This strategy has three subtypes: filter, wrapper, and embedding (Xu et al., 2021). In contrast, unsupervised approaches, such as principal component analysis (PCA) build low-dimensional feature representations by combining the original features in linear or non-linear ways without requiring the training label (Kim and Na, 2018; Xu et al., 2021).

#### Model training

The model and a suitable training method are chosen depending on the learning goal and data requirement. The hyperparameters that determine the model’s architecture (e.g., number of neurons, activation function, batch size, etc.) are then optimized for optimum performance, model generalization, and loss function reduction (Kim and Na, 2018; Xu et al., 2021). Hyperparameter tuning/optimization is the process of determining the optimal combination of hyperparameter values to get maximum data performance in an acceptable amount of time (Rojas-Domínguez et al., 2017). Hyperparameters differ amongst models and must be established before entering the training phase since they do not change and are not learned during training. Unfortunately, there is no mechanism to determine “what is the best approach to setting the model’s hyperparameters to minimize loss?” Thus, research and experiment are used to find the optimal option. In general, this process entails the following steps: defining a model, determining the range of possible values for all hyperparameters, sampling hyperparameter values by any of the different techniques to search for the ideal model structure, such as GridSearchCV and RandomizedCV, determining prediction error and other evaluation criteria to evaluate the model (Ali et al., 2021; Eslami et al., 2021). The prediction error is computed by applying the loss function to the expected value and the underlying truth. The choice of loss functions depends on the nature of the problem and the desired outcome. Mean squared error and mean absolute error are widely used in regression problems, while cross-entropy loss is utilized for classification (Eslami et al., 2021).

#### Model testing and performance evaluation

The model usually performs well in the training phase, but generalization requires further investigation in the testing phase. Test data should not be used during the training phase to avoid bias. Since most clinical data contains small samples that may lead to an insufficient model for training, unbiased cross-validation (CV) is frequently used to validate the model’s effectiveness and assess the data’s predictive capability. K-fold CV is a common validation method. To use it, the data set is divided into K subsets (also called folds) and used k times. The training process is initially performed on the K-1 subset, saving the remaining subset for later use as a test set and ensuring that the test and training sets do not overlap throughout each iteration (Xu et al., 2021).

Common confusion matrix-based quantitative measures of model performance are accuracy (ACC), sensitivity (Sen), specificity (Spe), positive predictive value (PPV), and negative predictive value (NPV). Whereas positive samples are autistic individuals and negative samples are healthy controls (HCs), true positive (TP), and true negative (TN) rates refer to the number of correctly classified positive and negative instances, respectively, while false positive (FP) and false negative (FN) rates refer to the number of incorrectly classified positive and negative instances, respectively (Uddin et al., 2017; Mostapha, 2020).

In statistics, accuracy is the proximity of repeated measurement results to the true value. It is also known as “diagnostic effectiveness” (Kong et al., 2019). The proportion of ASD disorders that were correctly diagnosed is referred to as sensitivity. “Specificity” is the proportion of typical developmental people whose ASD disorder was precisely excluded (Mostapha, 2020). The PPV of a test answers the question: “How likely is it that a patient who provided a positive test result has ASD?” While the NPV of a test provides an answer to the question: “How likely is it that a patient who gives a negative test result will not have ASD?”

Each metric describes a different aspect of the model’s power (Xiao et al., 2017). The receiver operating characteristic (ROC) curve is also widely used (Xu et al., 2021). This graph depicts the true positive rate (Sen) vs. false positive rate (1-Spe). The ROC curve is used to establish the appropriate cut-off point for both specificity and sensitivity. All possible combinations of Spe and Sen achievable by varying the test cut-off value can be summed up by utilizing the area under the receiver operating characteristic curve (AUC) parameter. AUC, or the area under the ROC curve, can never be more than 1, and the bigger it is, the more accurate the test is (Li H. et al., 2018). The F1 score is calculated by averaging the PPV and Sen scores. Therefore, this score takes both FP and FN into account (Panja et al., 2018; Wang et al., 2021).


(1) $$\text{ACC}=\frac{T\,P}{T\,P+T\,N+F\,P+F\,N}\times 100$$

(2) $$\text{Sen}=\frac{T\,P}{T\,P+F\,N}\times 100$$

(3) $$\text{Spe}=\frac{T\,N}{T\,N+F\,P}\times 100$$

(4) $$\text{PPV}=\frac{T\,P}{T\,P+F\,P}\times 100$$

(5) $$\text{PPN}=\frac{T\,N}{T\,N+F\,N}\times 100$$

(6) $$\text{F1}-\text{score}=\frac{2*T\,P}{2*T\,P+F\,P+F\,N}\times 100$$

### Common conventional machine learning and deep learning algorithms in autism spectrum disorder diagnostic research

**FIGURE 6:**
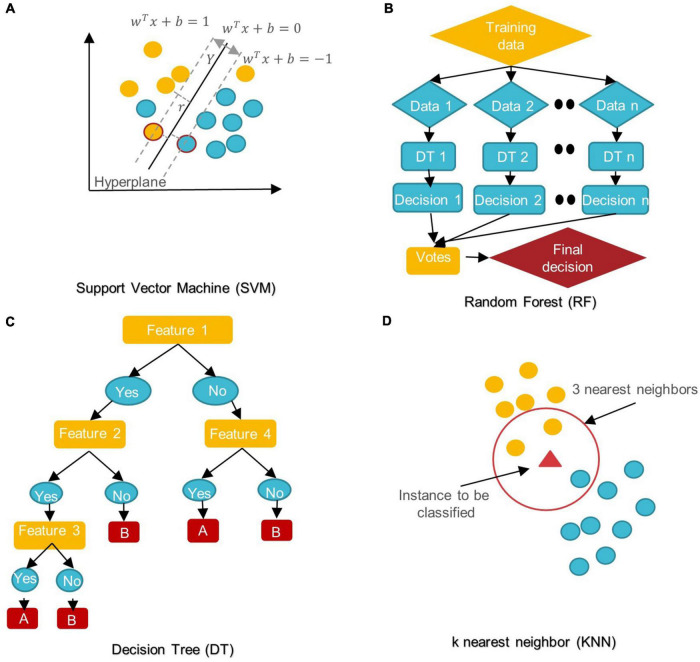
Schemes of conventional ML algorithms commonly used in MRI-based studies to diagnose ASD **(A)** SVM: support vector machine; **(B)** RF: random forest; **(C)** DT: decision tree; **(D)** KNN: k nearest neighbor.

In the last 5 years, several ML/DL models have been used in ASD research. Support Vector Machine (SVM), Random Forest (RF), Decision Tree (DT), Logistic Regression (LR), Naïve Bayes (NB), Boosting, and k-Nearest Neighbors (KNN) were among the most popular conventional ML algorithms (see Figure 6).

SVM aims to find the best decision boundary that increases the margin between classes in a high-dimensional space (Cortes and Vapnik, 1995). In SVM’s final discrimination function, only the data points (support vectors) closest to the hyperplane control the movement of the hyperplane that splits the data (Xiao et al., 2017). The kernel tricks of SVM can handle nonlinear classification, but they make the model harder to interpret (Panja et al., 2018). SVM is not influenced by outliers and is not sensitive to overfitting, but it is not optimal for a large number of features (Cortes and Vapnik, 1995; Xiao et al., 2017).

DT is a rooted directed tree with a flowchart-like structure that depicts the different consequences of a set of decisions. A DT contains branches that represent the data set’s features and leaf nodes that represent the outcome or decision (Liu et al., 2020). DT has good interpretability as it can approximate complex decision areas through a set of straightforward decision-making rules (Xu et al., 2021).

RF is made up of DT ensembles. RF uses random sampling with replacement (bootstrapping) to create many DTs during training. The forest is determined by the majority vote of the trees; hence, RF may give more accurate predictions than learning with a single DT (Uddin et al., 2017; Yin et al., 2020).

LR is a probabilistic method for estimating the statistical importance of features. Its purpose is to determine the values of the parameters that reflect all input variables. LR employs a logistic function in its most basic form to represent a binary dependent variable. It may also be modified to simulate many event classes (Uddin et al., 2017; Liu et al., 2020).

NB classifiers are based on Bayes’ theorem with high predictor independence. The NB classifier assumes that the effect of a predictor (x) on a particular category (c) is independent of the other predictors’ values. Despite its simplicity, NB classifiers often outperform more complex classification methods, especially for large data sets (Xiao et al., 2017; Bilgen et al., 2020; Chen T. et al., 2020).

In KNN, the data is categorized into several specified groups. It is carried out in such a way that all data points within the group are classified as homogeneous or heterogeneous when compared to data from other groups (Dekhil et al., 2021).

The boosting algorithm is an ensemble algorithm that transforms weak learners into strong ones. Weak learners have a weak connection to correct categorization. A strong learner is closely related to true classification (Bilgen et al., 2020).

**FIGURE 7:**
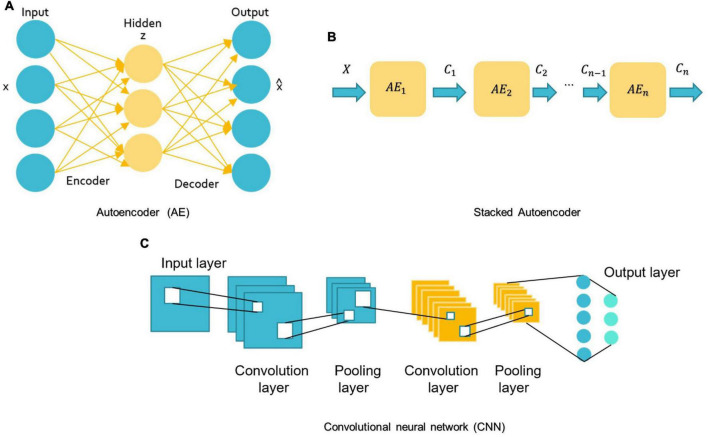
Schemes of DL algorithms are commonly used in MRI-based studies to diagnose ASD. **(A)** AE: autoencoder; **(B)** Stacked Autoencoder; **(C)** CNN: convolutional neural network.

For DL, there are several models such as Convolutional Neural Networks (CNN), deep generative models [e.g., Autoencoders (AE), Deep Belief Networks (DBN), and Generative Adversarial Networks], Multi-layer Perceptron (MLP), Recurrent Neural Networks (RNN), and Graph Convolutional Networks (GCN) that have been employed in various areas like computer vision, natural language processing, and speech recognition (Zhang L. et al., 2020; Khodatars et al., 2021). The first four were mostly used in reviewed ASD studies (see Figure 7).

MLP belongs to the class of feedforward neural networks with the same number of input and output layers but may have multiple hidden layers. MLP forward data from one layer to the next after linear and non-linear transformations (Libero et al., 2015; Mellema et al., 2019).

A basic AE requires two networks: an encoder network E and a decoder network D (see Figure 7A; Mostapha, 2020). The first network encodes the input data x into the low-dimensional space z, which is then used to decode and reconstruct the x data (Mostapha, 2020). Various types of AE, including contractive, sparse, and denoising AEs, were used in research for dimension reduction and to investigate the highly discriminative representations from neuroimaging data, but the spatial structure of data is often discarded (Kong et al., 2019). Stacking AEs, such as the stacked sparse AE (SSAE) is possible (see Figure 7B). Stacked AEs can learn faster than a single autoencoder (Zhang L. et al., 2020).

On the other hand, CNN can leverage the spatial information of sMRI data and deal with complex image processing problems. A standard CNN has multiple layers that process and extract features from data, as shown in Figure 7C. A CNN has many convolutional layers with multiple filters to perform the convolution operation. A filter (also called a kernel) determines the presence of certain features or patterns in the input. Next comes the Rectified Linear Unit to perform operations on the elements and produce a feature map (or activation map). The feature map is then fed to a pooling layer. Pooling is a down-sampling process that reduces the dimensions of a feature map. This makes the learning process relatively less expensive. Max and average pooling are the most widely used pooling techniques. The pooling layer flattens the 2D arrays of the pooled feature vector to create a single long vector by flattening it. The fully connected layer comes last, and it contains some hidden neural network layers. This layer classifies the image into different categories (LeCun et al., 2010; Ozonoff et al., 2011).

In the context of sequential data, RNNs are particularly beneficial since each neuron may retain the result of the previous stage in its internal memory and pass it to the current stage as input (Dua et al., 2020). This means RNNs can capture long-term relationships between input symbols (Mittal and Umesh, 2021). But training these RNNs is very costly (Dua et al., 2020). Moreover, early RNNs had simple recurring layers but they had a vanishing gradient problem and didn’t work for long data sequences (Dua et al., 2020). Long-short-term memory (LSTM) is the most prevalent architecture of RNNs used to solve these problems (Eslami et al., 2021; Quaak et al., 2021).

## Highlighted research

Recent advances in neuroscience and brain imaging and their combination with ML techniques using the methods described above allow us to understand the brain and the different regions implicated in ASD and their interaction. In this section, first, we summarize recent research on possible sMRI-based ASD biomarkers. Part 2 includes studies that only used conventional ML algorithms. Then we look at studies that benefited from DL algorithms.

### Identification of the potential autism spectrum disorder biomarker from structural magnetic resonance imaging

#### Whole brain volume and cortical lobes

Although the etiology of ASD remains unclear, for decades, an abnormally rapid increase in head circumference has been observed in some children with autism (Kanner, 1943; Pagnozzi et al., 2018). In light of the substantial correlation between head circumference and total brain volume (TVB), various volumetric studies have studied brain volume as a quantitative measure retrieved from sMRI (Pagnozzi et al., 2018). Atypical brain development in infancy may be used as an early ASD biomarker. Based on research by Hazlett et al. (2012), ASD children aged 2–4 had a larger brain than their peers, and in the next study (Hazlett et al., 2017), showed that ASD’s high-risk family members (HR) (Those who had a sibling with autism) at 6–12 months of age showed significantly higher cortical surface area (SA) growth compared to HCs, followed by an enlargement of TVB at 24 months of age as correlated with the severity of social autism. The SA growth rate increased mainly in the left and right middle occipital gyri, right lingual gyrus area, and right cuneus (Hazlett et al., 2017). Differences in the right occipital lobe are consistent with other studies (Irimia et al., 2018; Landhuis, 2020) that explain visual perception differences between ASD patients and HCs. This appears to correlate with a report by Irimia et al. (2018) that discovered the ASD group had higher areas and connectivity densities in the cuneus, occipital lobes, and the superior and transverse occipital sulci than the HC group. The ventral frontal lobe of ASD patients and HCs differs significantly (Irimia et al., 2018). The superior temporal gyrus curvature appeared smaller in ASD males than in ASD females, consistent with other results on ASD sex differences and their memory processing ability (Xiao et al., 2017) findings. Some studies link ASD to biological sexual differentiation, which might also explain the disparity in reported brain volume anomalies (Hazlett et al., 2012; Irimia et al., 2018). Adult autistic brain GM/WM volumes increased differentially and decreased across distinct areas, in contrast to early childhood increases in global volume measures (Akhavan Aghdam et al., 2018; Gorriz et al., 2019). This may be due to autism’s heterogeneity and potential subtle structural impacts or VBM’s limits, as VBM is sensitive to many artifacts, such as brain structure misalignment, which may mislead statistical analysis (Pagnozzi et al., 2018). In Dekhil et al. (2020) reported that changes in the size and shape of the cerebral cortex leading to an altered arrangement of WM fibers and changes in GM/WM; this, on its part, is relevant to identifying circuit abnormalities associated with autism. In Akhavan Aghdam et al. (2018), some differences exist in GM volume, particularly in the frontal and temporal regions, hippocampus, caudate nucleus, or other parts of the basal ganglia, amygdala, as well as the cerebellum. These areas include most of the TVB. TVB or intracranial volume (ICV) are essential factors for volumetric analyses of the brain (Kijonka et al., 2020). TVB = GM + WM (Hazlett et al., 2017). ICV is the sum of TVB and CSF volumes (Gorriz et al., 2019).

#### Cortical shape

Cortical thickness (CT), SA, the gyrification index (GI), and the sulcal morphology of the cerebral cortexes are all ROIs when investing in volume changes connected to ASD (Pagnozzi et al., 2018). The observed differences among studies are accentuated by the lack of agreement in designating ROIs. The CT is the shortest distance between the GM/WM border and the pial surfaces (Xiao et al., 2017). SA is the surface area of the WM. To find the cortical volume, multiply the SA by the CT (Xiao et al., 2017). CT levels in various brain regions have risen or fallen in different studies (Grimm et al., 2015; Raamana and Strother, 2020).

Motivated by evidence that regional CT measures can indicate cortical maturation and cortical-cortical connectivity and that ASD is characterized by delayed development, some studies (Moradi et al., 2017; Zheng et al., 2019) have supported CT scores as an ASD biomarker. For example, in Moradi et al. (2017) authors demonstrated a positive association between Autism Diagnostic Observation Schedule (ADOS) test-derived symptom severity and CT measurements. They also noted that age could affect CT and that the severity of the disorder determines age-related change. Their results also revealed a greater importance of the right hemisphere for predicting ASD severity than the left hemisphere. In Itani and Thanou (2021), the CT was higher in ASD than HCs in all ROIs identified at work. A statistically significant difference is shown in the superior frontal gyri, bilateral middle temporal gyri, right pars orbitalis, right insula, entorhinal cortex, and left superior temporal gyrus. Irimia et al. (2018) revealed similar findings in CT of the temporal lobes and superior temporal gyri. In contrast, Xiao et al. (2017) indicate that the left hemisphere is superior in distinguishing ASD. The left caudal anterior cingulate, the left parahippocampal, the left pars triangularis, and the left precuneus all have the same predictive value for ASD patients in the left hemisphere. According to Xiao et al. (2017), with neuroimaging data, the thickness-based classification of ASD performs better than both volume-based and SA-based classification. Broca’s area contains a part of the inferior frontal gyrus known as the Pars triangularis that contributes to language and social interaction difficulties. The caudal anterior cingulate is part of the social brain and mirror system hypothesis and cognitive regulation of behavior, including working memory, attention management, and decision making. The parahippocampal gyrus is critical for memory encoding and retrieval (Xiao et al., 2017). Thinning of the cerebral cortex within this region may affect the neural basis for risk disregard in autistic people (Xiao et al., 2017). Self-relevant mental images are identified in the anterior part of the precuneus, with posterior regions implicated in episodic memory visuospatial imagery, episodic memory retrieval, and self-processing operations. In summary, disruption in those four areas may be connected to ASD social issues and repetitive behaviors.

#### Cerebrospinal fluid

CSF is a fluid that surrounds the entire surface of the brain and the spinal cord and flows between the brain membranes. Although CSF is not technically a part of the human brain, it circulates nutrition, removes waste items generated by cerebral metabolism, and protects the brain from harm. However, CSF volume may be an indirect indicator of tissue loss in brain regions (Pagnozzi et al., 2018) and thus may be an important biomarker of ASD-induced brain-related changes. In young autistic children, an abnormal elevation of CSF can also occur, including movement, communication, and ASD status (Shen et al., 2017; Mostapha, 2020).

#### Cerebellum

The prefrontal cortex and cerebellum (Gao et al., 2022; Wang et al., 2021) and the temporal cortex (Wang et al., 2021) help classify structural covariance brain networks. Basic conscious motions are physically and functionally linked to the prefrontal cortex, and its abnormality is associated with ASD’s emotional and social domain. Studies have indicated that the superior temporal gyrus and the medial temporal cortex have direct connections that promote the memory for sound detection.

In addition, the cerebellum is essential for cognitive functions, memory, emotion, and language (Gao et al., 2022). The ASD and HC groups differed significantly in the choroid plexus, cuneus, left putamen, and cerebellar cortex (Pinaya et al., 2019).

#### Hippocampus and amygdala

The medial temporal lobe houses the hippocampus and amygdala, two interconnected subcortical structures. The hippocampus helps develop associative, spatial, episodic, and declarative memory. Similarly, the amygdala is involved in emotion and fear control and the recognition of facial expressions (Pagnozzi et al., 2018).

The amygdala and hippocampus have been linked with ASD-related deficits, including social cognition, eye-gaze direction perception, and emotion (Li et al., 2019b).

Researchers found that the ASD group had significantly less parahippocampal volume than the HC group (Irimia et al., 2018). As for the interaction with sex, autistic females had a larger right parahippocampal gyrus volume than autistic males.

Utilizing ML, another study found that the hippocampus plays a role in ASD (Fu et al., 2021). By comparing their findings with previous work on Alzheimer’s disease, they conclude that the overall severity of ASD-related morphological changes in the hippocampus is less pronounced or that the abnormality is more distributed in hippocampus areas.

In Li et al. (2019b), ASD was associated with significant enlargement of the amygdala and CA1-3 of hippocampal volumes in the right and left hemispheres. CA1-3 expansion could represent upregulation, reinforcing fear of communicating with the environment or others.

#### Basal ganglia, thalamus, and other proximal structures

The basal ganglia (BG) are neurons, also called nuclei, located in the depths of the cerebral hemispheres of the brain. In addition to coordinating postural muscle movements, the BG is involved in many regular behaviors and routines, such as the grinding of teeth, eye movements, and emotion. Although few studies have examined the role of BG in ASD symptoms, structural and strategic evidence suggests that ASD is linked to subcortical regions, including BG (Sivapalan and Aitchison, 2014; Pagnozzi et al., 2018; Ke et al., 2020). According to Irimia et al. (2018), those with ASD exhibited larger areas and volumes in some limbic structures such as the cingulate gyrus and the pericallosal sulcus. The temporal lobe, corpus callosum, middle cerebellar peduncle, caudate, and cingulate nucleus were the most relevant regions identified for predicting ASD in Guo et al. (2021).

### Conventional machine learning-based autism spectrum disorder classification applications

Several researchers have developed ML algorithms employing sMRI (Moradi et al., 2017) or multimodal data (Eill et al., 2019) to diagnose ASD or uncover novel biomarkers for it (Table 2, summarizing all ML-based studies). SVM has been extensively evaluated, with ACCs ranging from 45 to 94% on various ASD datasets (Morris and Rekik, 2017; Squarcina et al., 2021). In addition, various additional conventional ML models, such as NB (Xiao et al., 2017), KNN (Yassin et al., 2020), AdaBoost (Bilgen et al., 2020), and RF (Devika and Oruganti, 2020), have been examined. With an ACC = 94.29%, SVM was the most accurate of the ASD classification models, despite having the smallest data set (*n* = 35) (Devika and Oruganti, 2020). Zheng et al. (2019) use SVM and a multi-feature network to differentiate between ASD and HC. Their ACC is 78.63%.

**TABLE 2:** Summary of 20 ML-based ASD classification studies.

References	Modality	Biomarkers	^#^Subjects	Age	Preprocessing tool	Method used	Dataset	Best acc	Limitation
Xiao et al. (2017)	T1-w sMRI	Regional CT, cortical volume, and cortical SA	ASD = 46 Persons with DD = 39	ASD: 27 ± 4 DD:28 ± 4 months	FreeSurfer	SVM; NB; RF	Private data	CT: 75.6%	Small sample size. Children with developmental problems are used as HCs, which may result in deviations in the results.
Moradi et al. (2017)	T1 sMRI	CT	ASD = 156, HC = 0	8–40 years	CIVET pipeline	SVM	ABIDE I	51%	Continuation in regression models is inaccurate. The domain adaption becomes more challenging as the number of shared sites increases.
Morris and Rekik (2017)	T1w sMRI	Morphological brain connectivity using a set of cortical attributes	ASD = 59, HC = 43	−	FreeSurfer	SVM	ABIDE I	61.76%	Unknown is the age range of the participants. Small sample size. No comparison with deep learning.
Soussia and Rekik (2018)	T1-w MRI	Morphological brain connectivity	ASD = 155, HC = 186	ASD: 16.9 ± 6.3, HC: 16.6 ± 6.1 years	FreeSurfer	Ensemble classifier, SVM	ABIDE I	Avg left Himesphere:57.9%, Avg right Himesphere:61.6%	Imbalanced data. Using Pearson correlation to examine the link between ROIs may have ignored the non-linear nature of the relationship. Not investigated is the link between revealed cortical regions and non-cortical regions. No feature selection approach was employed.
Irimia et al. (2018)	sMRI and DTI	Thickness, area, volume, and curvature of GM, WM connectivity density	ASD = 110, HC = 83	ASD = 12.74 ± 2.79, HC = 13.04 ± 2.95 years	LONI Pipeline, TrackVis, FreeSurfer	SVM	Private data	93.26%	The models are lacking in transparency.
Eill et al. (2019)	fMRI, sMRI, and DWI	ROI-based FC and set of anatomic features	ASD = 46, HC = 47	13.6 ± 2.8 years	FreeSurfer, FSL and AFNI	Conditional random forest	Private data	CRF on top 19 variables: 92.5%	Small sample size. In tiny samples, cohort effects cannot be ruled out.
Zheng et al. (2019)	T1 sMRI	Seven morphological features (e.g., CT, SA, GM, Local gyrification index, sulcus depth, gyrus height), and elastic network	ASD = 66, HC = 66	ASD = 27 ± 8, HC = 27 ± 7 years	FreeSurfer, and SPM12	SVM	ABIDE I	78.63%	Only high-functioning ASD adults. The characteristics of participants vary considerably. In addition, the absence of key areas, such as the amygdala, may have significantly impacted categorization ability.
Gorriz et al. (2019)	T1 sMRI	GM; WM	HC = 60 ASD = 60	18–49 years	SPM12 and CAT12 toolbox	SVM	Private dataset	GM:69.47%, WM:66.16%	A small sample size.
Graa and Rekik (2019)	T1-w sMRI	Multi-view morphological brain networks based on the maximum principal curvature, the CT, the sulcal depth, and the average curvature.	ASD = 50, HC = 150	ASD mean age = 18.14 years, HC mean age = 17.91 years	FreeSurfer	SVM	ABIDE I	Left Hemisphere −4 views: 60%, Right Hemisphere −4 views: 59.5%	A small sample size consisting only of men. Utilizing a supervised classifier inhibits the framework’s scalability.
Bilgen et al. (2020)	T1-w sMRI	Cortical morphological networks	ABIDE I: ASD = 100 , HC = 100	−	FreeSurfer	Voting Classifier, Bagging Classifier, RF, AdaBoost, NB, Gradient boost, XGBoost, LR, SVM, DT, LDA, KNN, Quadratic Discriminant Analysis	ABIDE I and private dataset	1st team:70%	Only conventional ML models. No cross-validation is used.
Devika and Oruganti (2020)	sMRI (T1 +T2)	Three cortical measures CT, CA, and Cortical GM Volume	ASD:20, HC:15	1–4 years	FreeSurfer	LDA, RF, and SVM with Radial Bias Function (SVM-RBF)	ABIDE II	Without over-sampling (SVM and CA:94.28, SVM and CT:92.86, RF and CGMV :94.29) With over-sampling (SVM and CA:94.29, SVM and CT:94.28, RF and CGMV :94.29) SVM-RBF and CA:94.29	Very small dataset. Insufficient discussion of the study results. Each feature is analyzed independently from the others.
Chen T. et al. (2020)	sMRI	3D HOG	ASD = 119 , HC = 131	5.2–34.8 Years	MRIcron, SPM12	NB + SVM	4 datasets from ABIDE II	Each dataset had an AUC of at least 75%, with the greatest AUC of 0.849 occurring at the ETH location.	Heterogeneous datasets. The contribution of features to the classification result is the same whether they are 0 or 1.
Raamana and Strother (2020)	sMRI	CT-based networks	ASD = 100 HC = 100	ASD: 17.27 ± 7.68, HC: 15.82 ± 5.93 years	FreeSurfer	SVM	Preprocessed ABIDE I	AUC = 0.6	The SVM classifier alone was utilized. There was just one atlas used. Dataset is small and unbalanced. Only AUC is utilized as a performance metric.
Yassin et al. (2020)	T1-w sMRI	CT, SA, and subcortical features	Schizophrenia = 64, ASD = 36, HC = 106	Schizophrenia: 14–60 years, ASD: 20–44 years, HC = 16–60 Years	FreeSurfer: recon-all pipeline + Enhancing Neuroimaging Genetics	6 Classifiers, including, SVM, DT, AdaBoost, RF, KNN, and LR	Private data	Multiclass classification: LR using CT features = 69. Binary classification: ASD and schizophrenia all classifiers performed well (70 ≥)	ASD patients were only males. Small sample size.
Itani and Thanou (2021)	rs-fMRI +sMRI	Temporal and functional connectivity	ASD = 201, HC = 251	6–18 years old	C-PAC pipeline	DT	Preprocessed ABIDE I	74.8 ± 9.5%	Small and heterogeneous sample. A simplistic definition of brain topology.
Squarcina et al. (2021)	T1 sMRI	Regional CT	ASD = 40, HC = 36	9.5 ± 3.4 years	FreeSurfer	SVM	Private data	84.2%	small sample size. No independent test data.
Fu et al. (2021)	sMRI	Surface morphological features of bilateral hippocampus	ASD = 364, HC = 381	6–34 years	FIRST tool from FSL	Ensemble classifiers (boosting, subspace, bagging) + DT	ABIDE I	GentleBoost: > 80%	Cannot visualize the selected features and reduces understandability of this pipeline. Only used patch-based features. High level of heterogeneity
Mishra and Pati (2021)	T1-w sMRI	40 surface morphometric features + phenotype information such as age, VIQ, and FIQ	ASD = 26, HC = 24	−	recon-all workflow of FreeSurfer	DT and RF	ABIDE I	RF and 10-fold Cross-validation :88%	No information about the participant’s age. Very limited sample size.
Yalçin and Rekik (2021)	Rs-fMRI + sMRI	Multimodal brain graphs	Rs-fMRI: ASD = 254, HC = 272, sMRI: ASD = 155, HC = 186	-	fMRI: SPM + rs-fMRI data analysis toolkit. sMRI: FreeSurfer	LDA and SVM	ABIDE I	Multimodal classification model in several nodes in template graph = 20 with depth-based alignment and soft correspondence: 53.73%	The model is unexplainable. It must establish an appropriate threshold value for each modality in hand.

sMRI, structural MRI; fMRI, functional MRI; rs-fMRI, resting-state functional MRI; DWI, diffusion-weighted imaging; DTI, Diffusion-tensor imaging; ASD, Autism Spectrum Disorder; HC, healthy control; DD, developmental delay; GM, gray matter; WM, white matter; CT, cortical thickness; SA, surface area; CA, cortical area; FC, functional connectivity; LR, Logistic Regression; SVM, support vector machine; KNN, k-nearest neighbor; DT, decision tree; NB, naïve bayes; RF, random forest; LDA, linear discriminant analysis; M, male; F, female; HOG, histogram of oriented gradients.

^#^Number of subjects.

Individual variations in ASD symptom severity require individualized therapy based on associations between brain structure and clinical assessments of ASD risk, such as the ADI-R (Xiao et al., 2017) and ADOS (Moradi et al., 2017; Dekhil et al., 2021). Despite their limited scope, these studies are still noteworthy. In one study, RF, NB, and SVM were applied to 46 participants with ASD and 39 participants with developmental delay (Xiao et al., 2017). This study employed many differentiating features to increase classification accuracy: CT, cortical volume, and SA. The RF model was the most accurate, derived from the CT of 20 significant brain regions. Moradi et al. (2017) estimated ASD symptoms using ABIDE data and ADOS severity scores generated from CT measures. The authors suggested a method for creating a common space within various datasets to reduce inter-site heterogeneity called “domain adaptation.” At 51%, this research had the lowest ACC rate across studies. Dekhil et al. (2021) created a personalized CAD system using sMRI, rs-fMRI, and ADOS. The system produces a report for each subject that highlights ASD-affected regions. RF utilizing only rs-fMRI data produced 75% ACC, sMRI data produced 79% ACC, and combining the two produced 81% ACC. In another work (Dekhil et al., 2020), only sMRI and fMRI features were used to research brain region changes between ASD and HC groups to present a CAD system to help target therapeutic interventions. The system achieved good ACC (sMRI 0.75–1.00; fMRI 0.79–1.00) on a relatively large population using KNN and RF models.

Most research uses binary categorization. Some articles have many experiments. Gorriz et al. (2019), for example, divided participants into four groups based on gender and condition to compare ASD and HC brains. All binary categories “MH vs. FH,” “FH vs. FA,” and “MH vs. MA” (MH: male healthy, FH: female healthy, FA: female autism, MA: male autism) were developed to study gender differences in the diagnosis of ASD using SVM. Also, this article shows an example of different applications where binary classification applications of MH, FH, FA, and MA estimates were made twice; each time a distinct feature, either WM or GM volumes, was used to see which one could be most distinct in diagnosing ASD.

Like most previous research (Irimia et al., 2018), it suffers from the over-aggregation of features on insufficient sample size. Using MRI and DTI data, the study evaluated the applicability of SVMs for studying the relationships between an ASD diagnosis and gender. They demonstrated excellent ACC, but their findings cannot be generalized.

Network neuroscience is mostly focuses on fMRI-derived or DTI-derived FC features, which may neglect inter-regional morphological changes (Chen et al., 2011; Heinsfeld et al., 2018). Morphological brain networks (MBNs) may simulate this morphological connection between ROI pairs, in which the link between two regions encodes their morphological difference (Bilgen et al., 2020). The study (Morris and Rekik, 2017) employed SVM to evaluate the connectivity of cortical MBN collected just from sMRI. By concatenating low- and high-order network features, the authors extract features that are novel but lack biological value. Soussia and Rekik (2018) applied SVM and ensemble classifiers to complex MBNs, which represent shape-to-shape relationships between pairs of ROIs. Each network is associated with unique cortical features such as sulcus depth, curvature, and CT. But they didn’t apply any feature selection strategy. These studies also used specific ML approaches, leaving a large spectrum of methods unexplored for detecting ASD. To address this, a Kaggle competition was held to develop a suite of ML algorithms for diagnosing ASD utilizing MBN (Bilgen et al., 2020). The efforts of 20 teams were evaluated based on preprocessing, dimensionality reduction, and learning models. The two highest teams achieved ACCs of 70 and 63.8% using a powerful clustering algorithm called gradient boosting. Eill et al. (2019) also used a conditional RF ensemble algorithm and reported a high classification ACC of 92.5%.

Fu et al. (2021) postulate that prior studies on ASD classification using large datasets had low accuracy rates because they only considered SBM as scalar estimates (e.g., CT and SA) and neglected geometric information between features. Their application of the GentleBoost ensemble classifier to surface features of the bilateral hippocampus of male participants with ASDs and HCs. achieved an 80% ACC.

It has also been established that feeding an RF classifier with MRI and personal characteristics data improves ASD classification (Mishra and Pati, 2021).

Gao K. et al. (2021) developed a method for predicting the disease in 24-month-old infants utilizing sMRI and an XGBoost model. Some investigations used a histogram of oriented gradients (HOG) to analyze the gradient information of the aberrant region within the medical image (Ghiassian et al., 2016). Chen T. et al. (2020) found ASD biomarkers in children using a two-level morphometry classification framework based on the 3D HOG approach and NB model. Four ABIDE II locations achieved 0.75 AUC.

### Deep learning-based autism spectrum disorder classification applications

Recently, DL approaches outperformed conventional ML methods and were hailed as a major AI accomplishment. Unlike conventional ML algorithms, DL can automatically extract hierarchical features from incoming data and intelligently categorize them inside the model (Mostapha, 2020). In medicine, DL frameworks have been used to classify ASD from sMRI data alone (Hazlett et al., 2017) or with other modalities (Dekhil et al., 2021; Table 3 a summary of studies on DL).

**TABLE 3:** Summary of 25 DL-based ASD classification studies.

References	Modality	Biomarkers	N participants	Age	Preprocessing	Method used	Dataset	Acc	Limitation
Hazlett et al. (2017)	sMRI	Regional SA, CT, sex, and volume of intracranial	ASD- HR = 34, HC = 145	6–12 months	AutoSeg, CIVET	3-stage DNN: SAE +SVM	NDAR: IBIS	94%	Small sample and multi-stages approach
Demirhan (2018)	T1-w sMRI	Four different feature sets of morphometric measures	ASD experiment: ASD = 325, HC = 325	17.9 ± 7.4 years	FreeSurfer	SVM, KNN, and ANN	For ASD: ABIDE I	SVM:52 ± 7%	Subjects under the age of 10 are not included. The ABIDE dataset required additional iterations and time for SVM to reach convergence.
Dekhil et al. (2020)	sMRI + fMRI	8 structural features, and FC	ASD = 561, HC = 521	Different ages	FreeSurfer	KNN, RF	ABIDE I	sMRI: 78–100 fMRI: 79–100	Neglect of ASD heterogeneity. Need to test different neurodevelopmental conditions with ASD.
Dekhil et al. (2021)	sMRI + rs-fMRI + ADOS report	Spatial features: cortical volume (CV), CT, SA, and FC	ASD = 72, HC = 113	ASD males’ mean age = 13.07 years, and females mean age = 13.53 years HC males’ mean age = 13.04 years, and females’ mean age = 12.81 years.	FreeSurfer	SVM, KNN, RF, NB, and ANN	NDAR	RF: 80.8%	Data from multiple sources were used, which may restrict their utility in constructing a customized medicine model. This research may only apply to adults with high-functioning ASD who are between the ages of 8–18.
Akhavan Aghdam et al. (2018)	rs-fMRI, sMRI	Regional-based mean time series + GM + WM	ASD = 116, HCs = 69,	5–10 years	SPM 8	DBN of depth 3 + LR	ABIDE I and ABIDE II	65.56%	Small sample size, Raw data is not used as input data due to high data dimensions and limited computer resources. The model is complex and consumes significant computational time and resources for the training phase
		Regional based mean time series + GM				DBN of depth 3 + LR		65%	
		Regional-based mean time series + WM				DBN of depth 3 + LR		62.5%	
		WM				DBN of depth 3 + LR		59.72%	
		GM				DBN of depth 3 + LR		63.89%	
		Regional-based mean time series + GM + WM				DBN of depth 2 + LR		63.03%	
		Regional-based mean time series + GM				DBN of depth 2 + LR		61.94%	
		Regional-based mean time series + WM				DBN of depth 2 + LR		63.89%	
		WM				DBN of depth 2 + LR		61.11%	
		GM				DBN of depth 2 + LR		63.06%	
Sen et al. (2018)	sMRI and fMRI	Structural textures and 45 FC features	ASD = 538, HC = 573	7–64 years	SPM8 and in-house MATLAB code	AEs +CNN+ linear SVM	17 sites from ABIDE I	64.31%	Current results are not yet clinically relevant. Only used imaging data.
Li G. et al. (2018)	T1 sMRI	Several patches were extracted from several discriminative landmarks	ASD = 55, HC = 209	24-months	In-house tool	Multi-channel CNNs	NDAR	76.24%	Small sample size. Only for 24-month age. One train/test splitting for cross-validation.
Kong et al. (2019)	T1-w sMRI	Connectivity features between each pair of ROIs	ASD = 78, HCs = 104	The average age is about 15 years old	FreeSurfer	DNN: SSAE	NYU from ABIDE I	90.93%	Only bi-level (ASD/HC) classification was performed. Small sample size, imbalanced classes.
Mellema et al. (2019)	sMRI and fMRI	15 different feature sets: FC Matrix + anatomical volumes	ASD = 418HC = 497	−	−	SVM, RNN, CNN, GCN, DT, LR, RF, MLP	IMPAC	MLP: AUC = 80%	Sensitivity, and specificity metrics have not been evaluated.
Mostapha (2020)	sMRI	CT, SA, Shape Complexity Index and EA-CSF	ASD = 38HC = 149	6-months	FSL-BET, CIVET, and ANTs	MLP + CNN	NDAR: IBIS	89.7%	Small sample size
Ke et al. (2020)	sMRI	Different structural features	YUM:40 high SCQ, 33 = low SCQ. ABIDE: ASD = 946, HC = 1,046.	ASD = 29.4 ± 11.6 HC = 30.1 ± 5.3 years	SPM8	(1)3D input +2D/3D CNN, (2)2D/3D input +2D/3D CNN+2D/3D STN, (3)3D input+2D/3D CNN+ 3D STN +RNN, (4)2D/3D input +2D/3D CNN+2D/3D + CAM, (5)3D input+ RAM	Private data (YUM), ABIDE I +ABIDE II	ABIDE:2D Input + 2D CNN + 2D STN 59%. 2D Input + 3D CNN + 2D STN < 50%. 3D Input + 2D CNN + 3D STN 57%. 3D Input + 3D CNN + 3D STN 60%. 3D Input + 2D CNN + 3D STN + RNN 55%. 3D Input + 3D CNN + 3D STN+ RNN 56%. 2D Input + 2D CNN + CAM < 50%. 3D Input + 3D CNN + CAM 56%.	Inadequate accuracy to reach the level of clinical utility
Shahamat and Abadeh (2020)	sMRI	Normalized raw image	ASD = 500, HC = 500	7–64 years	FSL software	3D-CNN	ABIDE I	3D-CNN:70%, 3D-CNN + GABM:73%	Check the effect of different subsets of the regions that give different brain masks. Only one atlas is used to identify the knowledgeable brain regions; there need to try multiple atlases.
Ke and Yang (2020)	sMRI	Subcortical tissues	ASD = 30, HC = 9	−	−	DDPG-RAM, PER-RAM	NYU of ABIDE I	DDPG- RAM:85.6%, PER-RAM:87.4%	Only one site of ABIDE was used. Unbalanced dataset. The combination of DDPG and RAM necessarily increases several parameters, resulting in a decrease in processing speed.
Ferrari et al. (2020)	sMRI	296 brain morphometric features related to the global and subcortical features and the cortical features	ASD = 1,060, HC = 1,166	5–64 years	FreeSurfer	AEs + LR	ABIDE I and II	On 86 subjects: AUC = 0.79	The evaluation of the CI was limited only to 4 pairs of samples from ABIDE.
Zhang M. et al. (2020)	fMRI and sMRI	CT, SA, cortical volume, and singular values of fMRI connectome matrix	ASD = 537, HC = 590	17.01 ± 10 years	FreeSurfer and FSL	Discriminative learning + CNN	IMPAC2	69 ± 5.5%	Adopted only one type of CNN models
Rakic’ et al. (2020)	fMRI and sMRI	FC, and volumetric correspondences between cortical parcels’ GM volumes	ASD = 368, HC = 449	Mean age 14 years	FreeSurfer	Stacked AEs + MLP	ABIDE I	Combined data: 85.06 ± 3.52%	Exclude more than 100 subjects from ABIDE I dataset because did not meet the required preprocessing criteria.
Zhang and Wang (2020)	fMRI and sMRI	Brain surface morphometry and FC	ASD = 484, HC = 514	7–64 Years	FreeSurfer and ABIDE Preprocessed Connectome Project based on the C-PAC protocol	Geometric deep learning	ABIDE I	68.0 ± 03.8%	No cross-validation.
Gao K. et al. (2021)	T1w sMRI	Features from the segmentation and parcellation maps + sex information	IBIS: (ASD:52, HC = 195) ACE (ASD = 22, HC = 13)	IBIS (ASD = 24 ± 0.7, HC = 2 4 ± 0.89) ACE (ASD = 25 ± 1.5, HC = 24 ± 1.9) years	iBEAT V2.0 Cloud	CNN+SNN	NDAR: IBIS+ ACE	NDAR:91.5, ACE:82.9	Small number of 24- month-old subjects. Specific cortical surface features that accurately quantify early brain development were overlooked (i.e., mean curvature)
Ali et al. (2021)	sMRI	Set of morphological features	ASD = 505, HC = 530	6–64 years	FreeSurfer	LR, RF, SVM, AdaBoost, Passive Regression, and ANN	Preprocessed ABIDE I	ANN: 82% SVM:72%	No cross-validation. Un clear the final number of subjects in each class. Unclear which biomarkers contribute to models’ decision
Chen et al. (2021)	T1 sMRI and rs-fMRI	Brain networks	ASD = 481, HC = 526	−	C-PAC +Computational Anatomy Toolbox (CAT)	GCN for feature extraction+ MLP for classification	17 sites from ABIDE I	72.7%	Need to try using more ASD datasets to verify the robustness of the model.
Wang et al. (2021)	sMRI	Individual-Level MBN	ASD = 518, HC = 567	7–64 years	DRAMMS	Self-Attention Neural Network Classifier	ABIDE I	72.48%	Accuracy can be improved to be suitable for clinical use.
Gao et al. (2022)	sMRI	Individual-Level MBN	ASD = 518, HC = 567	7–64 years	DRAMMS	CNN	ABIDE I	71.8%	Not mention any harmonization process to solve the heterogeneity issue.
Tummala (2021)	T1 sMRI	Raw data	ASD = 112, HC = 102	ASD: 21 ± 8.7 HC:28.9 ± 8.5 years	FSL	SNN and Pre-trained ResNet50	ABIDE I	99%	Small sample size. A contrastive loss function was used. However, triple loss and quadrupole loss may perform better.
Guo et al. (2021)	(MRI including Axial T1, T2, FLAIR, and sagittal T1/T2) +ADC	MRI sequences	ASD = 151HC = 151 Test	= 45 1–6 Years	-	CNN based on ResNet 18 architecture	Private data	On validation set &DSM: 85.5% On test set &DSM: 84.4	It was a retrospective study. Need to explainable approach. No cross-validation. Only children younger than 7 years old. Without abnormalities in MR imaging. The mechanism of the FLAIR and ADC sequences for diagnosing ASD remains unknown.
Peng et al. (2021)	T1-w and T2-w sMRI	−	ASD = 289, HC = 180	6–12 months	−	GAN	NDAR: IBIS	69%	For training purposes, the technique requires paired longitudinal data from the same individual. The method is computationally expensive and requires high resources.
Gao K. et al. (2021)	sMRI	Cortical meshes and vertex-wise cortical shape metrics. In addition to sex	Human Connectome Project: female = 505, male = 606/ABIDE = 1,994 subjects	Different ages	FreeSurfer	Pretrained ResNet-50, pretrained DenseNet-121 and XGboost models	Human Connectome Project dataset, ABIDE I, and ABIDE	ResNet = 63.04% II	Cerebellum and subcortical regions are not involved in the analysis.
								DenseNet = 63.64%	
								ResNet (transfer) = 65.89%	
								DenseNet (transfer) = 65.59%	
								ResNet (2-stage) = 67.70%	
								DenseNet (2-stage) = 67.85%	

*sMRI, structural MRI; fMRI, functional MRI; rs-fMRI, resting-state functional MRI; ADC, apparent diffusion coefficient; SCQ, Social Communication Questionnaire; ASD, Autism Spectrum Disorder; HC, healthy control; GM, gray matter; WM, white matter; CT, cortical thickness; SA, surface area; Cx, cerebral cortex; CSF, cerebrospinal fluid; FC, functional connectivity; MBN, Morphological Brain Networks; ANN, artificial neural network; FFNN, feed forward NN; LR, Logistic Regression; SVM, support vector machine; CNN, convolutional neural network; FCNN, fully CNN; DT, decision tree; DBN, deep belief networks; RF, random forest; RNN, recurrent neural network; SAE, spares autoencoder; STN, Spatial Transformer Network; SNN, Siamese neural network; DFCN, deep fusion classification network; GCN, graph convolutional network; DDPG-RAM, Deep Deterministic Policy Gradient -RAM; PER, priority experience replay; M, male; F, female; ACE, Autism Centers of Excellence.

In Ali et al. (2021), a framework is presented that uses a recursive feature elimination method to select features and then trains linear, ensemble, and artificial neural network (ANN) models using them. ANNs enhanced categorization accuracy by up to 82%.

Several studies (Akhavan Aghdam et al., 2018; Sen et al., 2018; Mellema et al., 2019; Rakic’ et al., 2020) used sMRI and fMRI as inputs to the DL model. ASD disrupts FC between brain regions so many studies use FC neural patterns to distinguish ASD from controls. young autistic children were classified using the ABIDE I and II datasets and the DBN model (Akhavan Aghdam et al., 2018). The model achieved a maximum ACC (65.56%) combining three types of data (rs-fMRI + GM + WM).

Mellema et al. (2019) evaluated 12 classifiers using 915 IMPAC challenge dataset participants (Toro et al., 2018). These models comprise six non-linear shallow ML, three linear shallow, and three DL. For a fair comparison, the authors optimized each model’s hyperparameter using random search. To ensure that each model has the same training opportunity, random cross-validation was used. The dense feedforward network model achieves 80% AUC.

The reviewed studies employed various AE forms. In Sen et al. (2018), the authors utilized sMRI and rs-fMRI data to evaluate three ADHD and ASD learners. Learner 1 captures sMRI features using SAE-generated 3D texture-based filters and a CNN. Second learner computes non-stationary fMRI components. The final learner combines the structural and functional features of learners 1 and 2 and sends them to an SVM classifier, obtaining an ACC of 67.3% on ADHD data and 64.3% on ABIDE data, demonstrating that multimodal features can boost upset prediction accuracy.

Unlike fMRI, which is difficult to apply to infants, sMRI has gained interest for early ASD identification. sMRI is faster and contains infant-specific procedures, such as BCP (Howell et al., 2019; Gao K. et al., 2021). Deep generative algorithms were first utilized to predict ASD in infants using longitudinal data by Peng and others (Peng et al., 2021). Hazlett et al. (2017) developed a three-stage SAE model to diagnose infants with autism before the onset of behavioral signs. In contrast to most classification studies, which utilize cross-sectional data, a longitudinal dataset was used due to the relevance of a developmental approach to imaging, since ASD symptoms and implications may fluctuate over time (Lord et al., 2015). Despite the encouraging results in Hazlett et al. (2017), this multi-stage technique is inapplicable in clinical practice because it requires two scans at two different ages for tissue segmentation. An end-to-end and single scan-based method is used in Mostapha (2020). In Mostapha (2020), tissue segmentation was calculated automatically using a fully CNN.

MBNs that measure intracortical GM similarity are useful in the study of neurological disorders (Kong et al., 2019; Wang et al., 2021). The brain can be represented as a single view representation network or as a multi-view representation network (Graa and Rekik, 2019). Each view depicts a distinct morphological feature. Kong et al. (2019) used SSAE to learn low-dimensional brain connectivity patterns between each pair of ROIs from sMRI to build an individual brain network. Using only the 3,000 top F-scores features, the classifier had a 90.39% ACC. However, they used a small data set and did not depict potential biomarkers. The study (Gao et al., 2022) addressed this issue by identifying a biomarker using a Res-Net and gradient-weighted class activation mapping (Grad-CAM) on individual structural covariance networks. However, like most approaches to ASD diagnosis that have focused on features recovered from a separate ROIs, non-local relationships that are contradict brain network evidence have been ignored. In addition, Grad-CAM still has gradient saturation and pseudo-confidence issues (Wang et al., 2021). In Wang et al. (2021), a transformer-based DL architecture for more stable self-attention is presented; this self-attention DL model employs individual MBNs instead of raw MRI to identify ASD. Grad-CAM heat maps are hierarchical, like CNN’s simple-to-complex feature extraction algorithm (Gao et al., 2022). However, in Wang et al. (2021), the maps of self-attention coefficients in the first and second layers are similar, indicating a consistent diagnosis. In Leming et al. (2021), the authors verified their suggested approach for classifying individuals with ASD and age-, motion-, and intracranial-volume-matched HCs by feeding a CNN the symmetric similarity matrix from regional histograms of estimated GM volumes. They also used graph-theoretic metrics on output CAMs to determine CNN’s favorite categorization regions, focusing on hubs.

To address biases and outliers in training samples, a system based on two data selection tools was presented: an AE to discover outliers and a confounding index (CI) to identify sample variables that can complicate the learning process and mislead categorization (Ferrari et al., 2020). This technique doesn’t require costly computations or access to the true feature distribution. With the CI, the authors looked at how three categorical variables (gender, hands, and acquisition modality) and two continuous variables (age and FIQ) influenced the classifier. Gender, age, AM, and FIQ affected ASD/HC categorization, not hands.

Using 3D-CNN on the entire ABIDE dataset, an ACC of up to 70% was achieved (Shahamat and Abadeh, 2020). A genetic algorithm-based brain masking technique (GABM) was also developed to visualize the classifier’s function. The GABM approach enhanced the classifier’s final performance while making the model more easily interpretable.

Representations of features at the whole-brain level are not always effective in describing early structural changes in the brain. Therefore, several patch level features have been proposed, which are an intermediate measure between voxel level and ROI level to reflect the structural characteristics of the brain pathology diagnosis. In Li G. et al. (2018), the authors’ multichannel CNN with patch-level data expansion has been proposed to detect ASD in infants. The accuracy achieved was 24% better than 3D-CNN.

Graph convolutional networks (GCN) enable graph embedding by representing graph nodes, edges, and subgraphs as low-dimensional vectors. GCN can also learn graph topological structure information, which is essential for studying population brain networks (Chen et al., 2021). Research using GCN to classify autistic individuals under two distinct graph definition categories has been conducted (Parisot et al., 2018; Chen et al., 2021). The first establishes edges between subjects through phenotype information, including age, gender, and acquisition locations, together with the imaging-based node features (Parisot et al., 2018). The second type considers each subject as a graph (Chen et al., 2021). However, these two studies relied on single-modal MRI. To bridge the gap, Chen et al. (2021) developed an Attention-based Node-Edge GCN method that integrates sMRI and rs-fMRI data while concurrently modeling nodes and edges in graphs. Also, a gradient-based model interpretation technique was utilized to detect putative ASD biomarkers. Finally, an MLP model categorized the sequential feature maps. Zhang M. et al. (2020) suggests another multi-modal learning method that uses discriminative learning and CNN to classify ASD.

In several papers, multiple approaches have been compared. In Ke et al. (2020), an end-to-end training system was proposed using 14 distinct models that are mixtures of various network architectures, such as a static method (e.g., CNN), a sequential learning model (e.g., RNN), a Spatial Transformer Network (STN), a sequential feature learning model, or a feature visualization method (such as CAM). The 2D/3D CNN and RAM fared the best overall. Using both MRI (containing sagittal T1/T2 sequences, FLAIR, and T1/T2 axial sequences) and apparent diffusion coefficient (ADC) approaches, Guo et al. (2021) created a set of DL algorithms. The algorithms were trained using the ResNet-18 model, which had a “spatial channel” block to enhance feature identification. There are five single-sequence models, one dominant-sequencing model, and one all-sequencing model. The dominant sequence model had an ACC of 84.4%. Although the paper demonstrates an improvement in FLAIR and ADC sequencing performance, the sequencing process for diagnosing ASD remains unclear.

One study (Ke and Yang, 2020) developed a RAM-based approach for identifying ASD using sMRI data. To improve the convergence of the Policy Gradient approach used in conventional RAM, they developed a Deep Deterministic Policy Gradient-RAM (DDPG-RAM) model and a Gaussian sampling-based priority experience replay (PER) algorithm. This strategy increased ACC to 87.4% while enhancing accuracy and stability. The authors, however, used a small sample of 39 patients.

SNN is rarely employed in ASD research. S. Tummala applied SNN and a pre-trained ResNet model to 1,070 pairs of positive and negative images (Tummala, 2021). Table 3 provides a summary of the performance metric outcomes for each of the studies analyzed. Table 4 provides a summary of the assessment matrices utilized in ML and DL-based ASD investigations.

**TABLE 4:** Summary of evaluation matrices used in ML and DL-based ASD studies.

References	Test type	Accuracy	Sensitivity	Specificity	AUC	PPV	NPV	F1 score
ML-based ASD studies								
Xiao et al. (2017)	3-fold cross validation	75.6%	80 ± 3%	69.7%	0.8	−	−	77.8%
Moradi et al. (2017)	10-fold cross validation	51%	−	−	−	−	−	−
Morris and Rekik (2017)	leave-one-out cross validation	61.76	−	−	−	−	−	−
Soussia and Rekik (2018)	5-fold cross validation	Left Hemisphere: 57.1%						
		Right Hemisphere: 61%						
	10-fold cross validation	Left Hemisphere: 58.3%						
		Right Hemisphere: 62%						
Irimia et al. (2018)	10-fold cross validation	93.26%	97.17%	91.67%	−	−	−	−
Eill et al. (2019)	−	92.5	97.8	87.2	−	−	−	−
Zheng et al. (2019)	Leave-one-out cross-validation	78.63%	80.0%	77.27%	0.83	−	−	−
Gorriz et al. (2019)	Leave-one-out cross-validation	GM:69.47%	−	−	−	−	−	−
		WM:66.16%						
Graa and Rekik (2019)	Stratified 5-fold cross-validation	Left Hemisphere −4 views: 60%	−	−	0.6899	−	−	−
		Right Hemisphere −4 views:59.5%			0.6848			
Bilgen et al. (2020)	−	70%	72.5%	67.5%	−	−	−	−
Dekhil et al. (2020)	Customized cross-validation	sMRI 75–100; fMRI 79–100	sMRI 73–100; fMRI 78–100	sMRI 78–100; fMRI 79–100	Smri 0.79–1.00; fMRI 0.82–1.00	−	−	−
Devika and Oruganti (2020)	Leave-One-Out-Cross-Validation	Without oversampling: SVM and CA:94.28%						−−
		SVM and CT:92.86%	−	−	−	−	−	−
		RF and CGMV:94.29%	−	−	−	−	−	−
		With over-sampling SVM and CA:94.29%	−	−	−	−	−	−
		SVM and CT:94.28%	−	−	−	−	−	−
		RF and CGMV:94.29%	−	−	−	−	−	−
		SVM-RBF and CA: 94.29%	−	−	−	−	−	−
Chen T. et al. (2020)	10-fold stratified cross-validation	−	−	−	0.75 in each dataset	−	−	−
Raamana and Strother (2020)	Repeated nested split-half cross-validation	−	−		0.6	−	−	−
Yassin et al. (2020)	10-fold cross-validation	Multiclass classification: LR using CT features = 69%	−	−	−	−	−	−
		Binary classification (ASD and schizophrenia): All features +LR = 70%. All features +SVM = 75%. All features +KNN = 75%. Subcortical features +RF = 75%. CT features +Adaboost = 85%						
Itani and Thanou (2021)	Leave-one-out cross-validation	74.8 ± 9.5%	−	−	−	−	−	−
Squarcina et al. (2021)	Leave-one-out cross-validation	84.2%	80%	88.9%	−	−	−	−
Fu et al. (2021)	10-fold cross validation	83 ± 0.07%	80 ± 0.1%	85 ± 0.06%	–	84 ± 0.06%	82 ± 0.08%	−
Mishra and Pati (2021)	5-fold cross-validation	RF:86%	−	−	0.91	−	−	−
	10-fold cross-validation	RF:88%			0.90			
Yalçin and Rekik (2021)	5-fold cross-validation	fMRI brain network with non-linear similarity network fusion: 58.18%	49.1%	66.1%	−	−	−	−
		Morphological brain network with averaging method: 57.63%	42.72%	69.89%	−	−	−	−
		Multi-modal classification model in number of nodes in template graph = 20 with depth-based alignment and soft correspondence: 53.73%	45.68%	61.03%				
DL-based ASD studies								
Hazlett et al. (2017)	10-fold cross validation	94%	88%	95%	−	81%	97%	−
Demirhan (2018)	5-fold cross validation	52 ± 7%	−	0.54	−	−	−	−
Dekhil et al. (2021)	4-fold cross validation	80.8%	84.9%	79.2%	81.92%	−	−	−
Akhavan Aghdam et al. (2018)	10-fold cross validation	65.56%	84%	32.96%	−	−	−	74.76%
Sen et al. (2018)	5-fold cross validation	64.31%	60%	68.32%	−	−	−	−
Li G. et al. (2018)	10- fold cross validation	76.24%	−	−	−	−	−	−
Kong et al. (2019)	10-fold cross validation	90.39%	84.37%	95.88%	0.9738	−	−	−
Mellema et al. (2019)	3-fold stratified cross-validation	−	−	−	80	−	−	−
Mostapha (2020)	10-fold cross validation	89.7%	78.3%	92.5%	−	80.2%	95.2%	−
Ke et al. (2020)	10-fold cross validation	2D Input + 2D CNN + 2D STN 59%	−	−	−	−	−	−
		2D Input + 3D CNN + 2D STN ¡ 50%	−	−	−	−	−	−
		3D Input + 2D CNN + 3D STN 57%	−	−	−	−	−	−
		3D Input + 3D CNN + 3D STN 60%	−	−	−	−	−	−
		3D Input + 2D CNN + 3D STN + RNN 55%	−	−	−	−	−	−
		3D Input + 3D CNN + 3D STN + RNN 56%	−	−	−	−	−	−
		2D Input + 2D CNN + CAM¡ 50%	−	−	−	−	−	−
		3D Input + 3D CNN + CAM 56%	−	−	−	−	−	−
Shahamat and Abadeh (2020)	5-fold cross validation	3D-CNN :70%	−	−	−	−	−	−
		3D-CNN + GABM:73%	−	−	−	−	−	−
Ke and Yang (2020)	5-fold cross validation	DDPG-RAM:85.6%	93.2%	65.7%	0.830	87.7%	78.9%	−
		PER-RAM:87.4%	93.7%	69.9%	0.937	89.7%	80%	−
Ferrari et al. (2020)	10-fold cross-validation	−	−	−	0.79	−	−	−
Zhang M. et al. (2020)	8-fold cross-validation	0.690 ± 0.055	0.790 ± 0.049	0.689 ± 0.048	0.733 ± 0.051	−	−	−
Rakic’ et al. (2020)	Leave-one-site-out cross-validation	over 70% for all ABIDE I studies but the CMU center, where the accuracy was 60%						
	10-fold cross validation	fMRI +ensemble of classifiers: 74.90%	74%	76%				
		sMRI +ensemble of classifiers: 78.69%	78%	79%				
		Combined data + an ensemble of 5 functional and 5 structural data classification models = 85.06 ± 3.52%	81%	89%				
Zhang and Wang (2020)	−	0.680 ± 0.038	−	−	0.617 ± 0.044	−	−	−
Gao K. et al. (2021)	10-fold cross validation	NDAR :91.5%	86.5%	92.8%	0.91	−	−	−
		ACE :82.9%	81.8%	84.6%	0.86%			
Ali et al. (2021)	–	SVM:72% NN: 82%	−	−	−	−	−	−
(Chen et al., 2021)	10-fold cross validation	72.7%	67.8%	76.6%	–	–	–	–
		Avg :69.42%	–	–	–	–	–	
Wang et al. (2021)	−	72.48	75.81	68.09	0.74	−	−	0.7581
Gao J. et al. (2021)	10-fold cross-validation	71.8%	81.25%	68.75%	67%			0.6868
Tummala (2021)	5-fold stratified cross-validation	99%	−	−	−	−	−	0.99
Guo et al. (2021)		3D CSResNet-18 on Validation set: 85.5%	84.2%	86.8%	0.896	−	−	−
		3D CSResNet-18 on Test set: 84.4%	85.0%	84.0%	0.898	−	−	
Peng et al. (2021)	4- fold cross-validation	69.9%	−	−	0.671	−	−	0.694
Gao K. et al. (2021)	10-fold cross-validation	ResNet = 63.04%	52.40%	72.51%	0.6756	−	−	−
		DenseNet = 63.64%	55.80%	70.62%	0.6725	−	−	
		ResNet (transfer) = 65.89%	60.28%	70.90%	0.6996	−	−	
		DenseNet (transfer) = 65.59%	57.29%	72.99%	0.7018	−	−	
		ResNet (2-stage) = 67.70%	62.73%	72.13%	0.7199	−	−	
		3DenseNet (2-stage) = 67.85%	61.66%	73.36%	0.7237	−	−	

### Magnetic resonance imaging datasets

Listed below are the publicly available datasets in the reviewed publications (see Table 5).

**TABLE 5:** Summary of publicly brain MRI datasets used in ASD studies.

References	Dataset name	Date released	Num of images/classes	Link	Used in
Di Martino et al. (2014)	ABIDE I	2012	539 ASD (360 M,179 F), 573 non-ASD (403 M, 170 F) (Ages 7–64 years, mean age 14.7 years across groups)	http://fcon_1000.projects.nitrc.org/indi/abide/abide_I.html	Moradi et al., 2017; Morris and Rekik, 2017; Akhavan Aghdam et al., 2018; Demirhan, 2018; Sen et al., 2018; Soussia and Rekik, 2018; Gorriz et al., 2019; Graa and Rekik, 2019; Kong et al., 2019; Zheng et al., 2019; Bilgen et al., 2020; Dekhil et al., 2020; Ferrari et al., 2020; Ke and Yang, 2020; Ke et al., 2020; Raamana and Strother, 2020; Rakic’ et al., 2020; Shahamat and Abadeh, 2020; Zhang and Wang, 2020; Ali et al., 2021; Chen et al., 2021; Fu et al., 2021; Itani and Thanou, 2021; Mishra and Pati, 2021; Gao K. et al., 2021; Gao et al., 2022; Tummala, 2021; Wang et al., 2021; Yalçin and Rekik, 2021
Di Martino et al. (2017)	ABIDE II	2017	521 ASD (414 M, 73 F), 593 non-ASD (382 M,175 F) (Age range: 5–64 years)	http://fcon_1000.projects.nitrc.org/indi/abide/abide_II.html	Akhavan Aghdam et al., 2018; Devika and Oruganti, 2020; Chen T. et al., 2020; Ke et al., 2020; Ferrari et al., 2020; Gao K. et al., 2021
Payakachat et al. (2016)	NDAR	2016	In 2014: Data from over 77,000 subjects. Multimodal-MRI: 4,745 subjects.	https://nda.nih.gov/about.html	Hazlett et al., 2017; Li G. et al., 2018; Mostapha, 2020; Dekhil et al., 2021; Gao K. et al., 2021; Peng et al., 2021
Toro et al. (2018)	IMPAC	2018	1,150 subjects in the public set (601 HCs, 549 ASD). 1,003 subjects in the test set (591 HC,412 ASD)	https://paris-saclay-cds.github.io/autism_challenge/	Mellema et al., 2019; Zhang L. et al., 2020

ABIDE, Autism Brain Imaging Data Exchange Initiative; NDAR, National Database for Autism Research; IMPAC, Imaging Psychiatry Challenge: predicting autism.

(1) Autism Brain Imaging Data Exchange Initiative (ABIDE):

It has combined functional and structural neuroimaging data from several laboratories to further the knowledge of the neurological underpinnings of autism (de Belen et al., 2020). It has two collections: ABIDE I and ABIDE II.

(a) ABIDE I was co-constructed by 17 international sites. With this effort, a total of 1,112 records (sMRI + fMRI) were created for participants comprised of 539 autistic people and 573 non-autistic individuals (7–64 years old) (Di Martino et al., 2014).

(b) ABIDE II was created to enhance brain neural network discovery in ASD. It covered 19 international sites with 1,114 subjects’ records from 521 autistic people and 593 from non-autistic individuals for sMRI (5–64 years old) (Di Martino et al., 2017).

The ABIDE dataset has been criticized for being collected from different locations using different imaging devices that may affect the output. However, after investigating the confounding effects of scanners on images, it was found that robust results still exist. Furthermore, ABIDE offers a unique opportunity to analyze a large sample of females with ASD, as this was not possible with other datasets and is partly due to differential prevalence rates between males and females (Richards et al., 2020). The ABIDE site displays scan techniques, settings, and participants’ inclusion and exclusion criteria for each site (Guo et al., 2021).

(2) National Database for Autism Research (NDAR): It is a National Institutes of Health-funded research data repository (Payakachat et al., 2016). It offers neuroimaging datasets from different ages and modalities (Dekhil et al., 2021). The data comes from George Washington University and California University’s Center for Autism. NDAR’s imaging data is anonymized and linked to other records (diagnostic, behavioral, demographic, etc.) (Li G. et al., 2018). The Infant Brain Imaging Study (IBIS) and Autism Centers of Excellence (ACE) studies from NDAR were most utilized (Hall et al., 2012; Payakachat et al., 2016).

(3) Imaging Psychiatry Challenge: predicting autism (IMPAC): More than 2,000 participants submitted sMRI and fMRI scans. The general group has images of 1,150 people (601 HC, 549 ASD), 920 males, and 230 females. The test group consisted of 1,003 people (758 men and 245 females) (591 HC, 412 ASD). Participants were of various ages. sMRI was processed using FreeSurfer, and FSL describes gray matter volume, area, and thickness. fMRI is a time series taken from different atlases (Dukart et al., 2016; Tate et al., 2020).

## Discussion and limitations

ML-aided MRI classification has offered new psychiatric and neurological research possibilities. First, biomarkers can enhance behavioral-based diagnosis. Second, understanding the indicators can assist locate the defect and thus target it with medications and treatments. Third, biomarker testing on children and infants can help doctors treat and support them earlier and more effectively.

**FIGURE 8:**
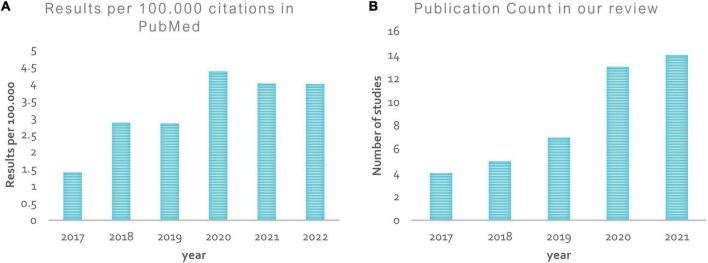
Publication by year. **(A)** Shows a rise in the number of papers published in the ASD diagnosis area from 2017 onward, according to the “PubMed by year”; **(B)** represents the number of papers published, reviewed here, by year.

Figure 8 shows a rise in the number of papers published in this area over time. From 2017 onward, the “PubMed by Year”^1^ project has generated a graph showing the number of articles published each year that meet the formula used to list previous studies in our review in over 26,000 journals around the world (see Figure 8A). However, the simple count is no longer a credible metric of research progress due to the growing literature. On the other hand, the graph in Figure 8B represents the number of papers published, reviewed here, by year.

**FIGURE 9:**
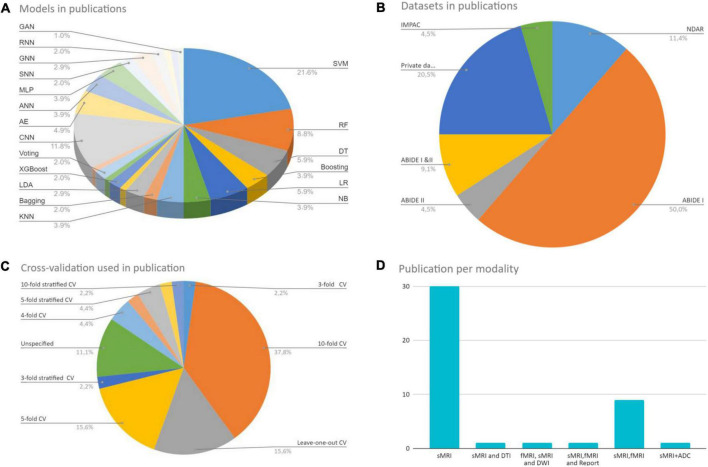
Reviewed studies analysis. **(A)** Shows a variety of ML and DL approaches used to diagnose ASD. **(B)** Shows a different set of CV techniques. **(C)** Shows the data sets used in the studies and their number. **(D)** Shows the imaging modality used to build the models.

ML/DL shows promise in neuroimaging-based ASD diagnosis despite its early use. All studies confirm structural and functional ASD anomalies. However, the current taxonomic literature’s inconsistency, especially in structural features, suggests it cannot capture disease diversity and should be interpreted cautiously. When evaluating the literature, research parameters may constrain assumptions about ASD with respect to population wide. Because of this, study results are rarely reproducible. The age of participants, ML algorithm type, behavioral variability, and total sample size all contribute to these disparities. It is unfair to compare a technique that tested 1,000 people and had low classification accuracy to one that tested fewer than 100 people and had high classification accuracy (Devika and Oruganti, 2020). Models with a small sample tend to be overfitted, resulting in a pattern susceptible to outliers and biases. Sadly, there is no universally applicable framework for algorithms and classifiers used in disease research. This is in line with the “no free lunch” principle (Tate et al., 2020), which states that no algorithm works better than another for all problems. Figure 9 depicts the large variance between studies. Figure 9A shows a variety of ML and DL approaches used to diagnose ASD.

In conventional ML, SVM is commonly used. Among the various DL architectures, CNNs were found to be the most popular, with the most promising results. Also, the AEs results were positive. Figure 9C shows the data sets used in the studies during our research and their number. The ABIDE dataset received the lion’s share. Figure 9B shows a different set of CV techniques. The last figure shows the imaging modality used to build the models, with single modality models being the most common. Because each research is unique, it may include a variety of MRI techniques and clinical data. The different methodologies and measurements used in the studies make direct comparison difficult.

Nonetheless, these studies can provide valuable data for future researchers. As shown in the analysis and Tables 2, 3, most research uses volumetric measures to distinguish between healthy and autistic brains. Studies have linked ASD-related structural abnormalities to the temporal, occipital, and frontal lobes. Several studies have found CT to be a critical ASD biomarker. The high dependence on FC patterns is also an fMRI main feature.

To date, sMRI-based biomarkers cannot replace clinical assessments in diagnosis, but they may alter therapy objectives and procedures. Inconsistently designating a biomarker that occurs in some patients but not others is a concern. Clinicians should educate families about their child’s biomarker without compromising determinism, because a biomarker may indicate an elevated potential for the disorder but is not necessary. AI-assisted predictive modeling can predict a disease before clinical symptoms appear. To establish the prediction power of early identified brain characteristics, infants must be sampled. A few studies applied ML to infant data (Hazlett et al., 2017; Xiao et al., 2017; Li G. et al., 2018; Mostapha, 2020). Hazlett et al. (2017) obtained a 94% ACC utilizing DL on 34 high-risk ASDs and 145 non-ASDs. Future studies should be longitudinal and sibling of high-risk individuals.

There has been a recent movement toward integrating diagnostic modalities such as fMRI, DWI, and sMRI. It may improve prognosis accuracy by using complementary information in the multimodal data (Eill et al., 2019; Chen et al., 2021). Only one study combined non-imaging data (such as demographic data and reports) with imaging data to enhance classifier predictability and interpretability (Dekhil et al., 2021). While this combination has been applied in studies of other diseases (Guo et al., 2015; Dukart et al., 2016), developing such ML frameworks for the automated diagnosis of ASD is advisable. Using several imaging modalities and multiparametric features does not always increase diagnosis performance (Akhavan Aghdam et al., 2018). In addition, recently developed unimodal MRI methods are comparable to state-of-the-art multimodal approaches (Shahamat and Abadeh, 2020; Zhang and Wang, 2020). Multimodal methods are more sensitive and give more information in general, but sMRI-based CAD is more appealing because it is cheap, and widely available in the clinic.

**FIGURE 10:**
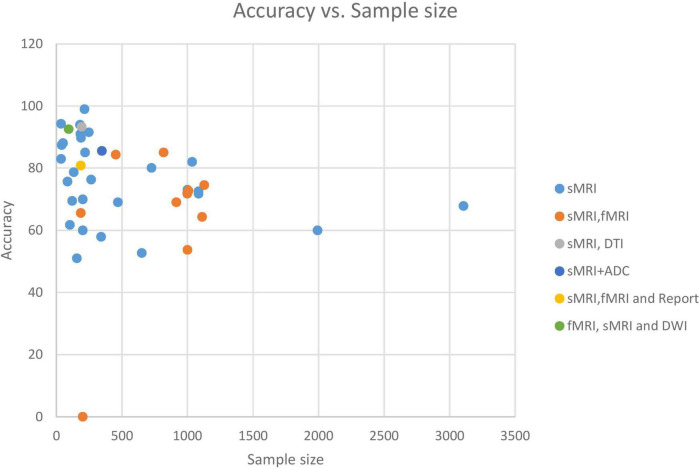
Shows relationships between the sample size and the accuracy of the studies.

Tables 2–4 and Figure 10 indicate that research accuracy reduces with more participants. Large populations have more clinical phenotypic heterogeneity. Moreover, data from many sites, such as ABIDE, can be hampered by heterogeneity due to differences in scanner types, data collection and processing techniques, demographics, and disease assessment. As a result, classifiers learn site-specific variables, not crucial data. Building precise and stable learning models from heterogeneous multi-site data is still difficult. “Domain adaptation” approach minimizes between-site heterogeneity. Also, preprocessing is required to remove subject-specific variability. In studies using a subset of multi-site datasets, heterogeneity is overlooked, leaving model performance in other datasets uncertain. To accurately diagnose ASD, these algorithms must be evaluated on various datasets. Increasing sample size implies more reliable results, as statistical significance should have enough power with a bigger sample. However, there are important non-statistical considerations, such as relative gaps in age and sex representation in studied sample populations. Moreover, negative results are likely to be overlooked in the articles due to a bias toward reporting positive results, referred to as the “file drawer problem.” Only (Ferrari et al., 2020; Ke et al., 2020) articles have shown how ML can be used to categorize ASD patients using the two large ABIDE databases. Here, too, we must point out that only limited dataset repositories are available to the public (with only two classes: ASD and HC or non-ASD) and are adopted in most ASD research.

According to our research, ensemble classifiers outperform individual classifiers. For example, when training on a small dataset, several hypotheses can produce the same accuracy. Averaging these hypotheses may help the ensemble solve this problem. Second, ensemble learning decreases the learned model’s sensitivity to the limited training data by merging many classifiers, resulting in better generalization of the trained model. Finally, using many linear classifiers rather than one non-linear classifier allows for linearly inseparable data while keeping the model simple (Fu et al., 2021).

Small training data sets promote “overfitting” in most experiments. The models’ complexity exacerbates the issue. Because ASD repositories had limited MRI data, researchers used various ways of preventing overfitting. Regularization (L1/L2, Drop-Out, and Batch Normalization) decreases model complexity (Soussia and Rekik, 2018; Mellema et al., 2019). Cross-validation also has been used in several research to prevent overfitting when the model’s complexity or dataset size cannot be modified (Moradi et al., 2017; Sen et al., 2018). Here, one study used a single split of training and testing, which resulted in an overly optimistic result that confuses comparisons with other studies (Li G. et al., 2018). Because the ideal practice for model generalization is to utilize an independently gathered dataset as the test set, reporting a leave-one-out CV is acceptable, as each site represents a separate dataset. This is not currently standard practice, as most of the research has used data from large multi-site databases, but only a few have reported LOOCV (Gorriz et al., 2019). Due to the imbalance between classes, Synthetic Minority Oversampling Technique (SMOTE) is utilized in conjunction with LOOCV because of the imbalance between them (Devika and Oruganti, 2020). When the sample size is modest, this method of validation is appropriate.

Another drawback relates to the feature extraction time because each participant must be analyzed separately. Usually, neuroimaging data is stored in high-dimensional space; for example, a 60 × 60 × 60 3D image can yield 216,000 features. As a result, classifiers may be trained on small datasets; thus, increasing the likelihood of ML overfitting. Therefore, automated feature extraction is desirable. To address this, most recent works leverage off-the-shelf pipelines or preprocessing tools like FreeSurfer to extract features before feeding them to ML models and reduce computing overhead (Raamana and Strother, 2020). Pipelines also enable method comparison (Khodatars et al., 2021). Compared to adult MRI pipelines, just a few infant MRI pipelines exist now, such as infants such as Infant FreeSurfer (Wang et al., 2018; Zollei et al., 2020).

Several strategies have been used to reduce the number of input dimensions, retain relevant information by assessing each dimension’s value, and control the classifier’s complexity to avoid overfitting (Gorriz et al., 2019). Existing methodologies for reducing the dimensionality of features include feature extraction, feature selection, and sparse learning methods. F-score (Devika and Oruganti, 2020; Fu et al., 2021), Recursive Feature Elimination (Ali et al., 2021; Fu et al., 2021), PCA (Irimia et al., 2018), greedy selection (Squarcina et al., 2021), and AEs (Sen et al., 2018) are examples of feature selection techniques.

Another issue in ML is a class imbalance that makes “accuracy” meaningless, which means disproportionate samples for each category, such as in medical data sets where controls often outnumber patients. Due to the low rate of ASD, models tend to favor the majority group, making it hard to improve accuracy while reducing false positives and negatives (Eslami et al., 2021).

The participants’ sex representation in the literature is remarkably unbalanced. Until recently, most ASD classification algorithms used only male (or mostly male) samples. The relative absence of females hinders our understanding of ASD in females. A few studies (Irimia et al., 2018; Gorriz et al., 2019) trained and evaluated the classifiers using comparable sample of males and females. However, there is emerging evidence that biological sex differences impact ASD risk and contribute to the reported 4:1 male bias in diagnosis. Including sexual information in the model was one way to find differing male and female development patterns. K-fold validation, re-sampling the training set by over-sampling the smaller minority group, under-sampling the larger majority group have been used to mitigate the effect of the majority class on the final prediction (Sen et al., 2018).

Age matters with determining ASD anatomical alterations. The current age for the studies reviewed here ranges from 6 months to 64 years. This makes broader inferences more difficult because of the brain’s plasticity and developmental potential through the first years of life. This issue may be addressed in typically developing children and through comparisons of changes in anatomy with age in ASD. It would be beneficial to gather more information on children under the age of eight as they are not well-represented at this time. This age range represents a pivotal time as children are first being diagnosed, interventions and treatments are being prescribed, and the brain is undergoing major developmental changes. It is worth noting that most studies thus far have examined ASD in older individuals diagnosed at a younger age and who have undergone years of treatment, and education.

Finally, one should carefully consider the ASD subgroup tested for a study before making larger inferences, as studies generally include higher-functioning ASD individuals with relatively normal IQ scores.

## Future direction

There is still room to improve existing research studies to increase diagnosis accuracy, uncover robust biomarkers, and support clinical evaluation of ASD, which can help guide treatment decisions and improve long-term outcomes. Here we highlight some upcoming trends in this field.

(1) Potential biomarkers must meet certain criteria before they can be used in clinical settings. Biomarkers must be distinct to each ailment, not a general disease sign, and symptoms can’t imply anything without them (Du et al., 2018). To achieve the classifiers’ specificity, many samples with various diseases must be evaluated. Also, studies on autism-related brain changes identify regions scientifically associated to autism and others with unrecognized roles or little implications. To improve ASD diagnosis and gather solid biomarker information, undiscovered regions must be studied using big data and 3T or 7T scans.

(2) With the increased production of data, diagnostic imaging has begun to take shape entirely in “big data,” which is defined in a healthcare context as “biological, clinical, environmental, and lifestyle information” collected from individuals to large groups about their health and state of health at one or more points in time (Sejdic and Falk, 2018). Finding ways to analyze big data applications and manage security risks is an important future direction. Cyber-attacks target healthcare data repositories and organizations to access, change, delete or steal sensitive data. Every 39 seconds, a vulnerability on the Internet is exploited to start malware attack (Kumar et al., 2020). The dark web’s high cost of healthcare information makes it a popular target for hackers (Kumar et al., 2020). Another problem with big data is that it contains a huge amount of explicit and implicit knowledge that adds great value to healthcare. However, effective search for and use of this knowledge faces many challenges, such as developing healthcare knowledge management systems and possibly supporting them with ML algorithms to derive systematic knowledge of different data at a higher level to support diagnosis and treatment (Phan et al., 2022; Dicuonzo et al., 2022).

(3) As clinical scientists and mental health experts remain worried about AI interpretation concerns, ML/DL algorithms must be made more transparent and trustworthy by supporting both human and machine decision-making. Explainable AI (XAI) has arisen as a critical international issue when employed in medical decision-making (Ozonoff et al., 2011; Pinaya et al., 2019). XAI investigates the rationale behind the decision-making process, explains the system’s benefits and limitations, and speculates on its future behavior (Yang et al., 2022). A typical XAI feedback loop includes training, quality assurance, deployment, prediction, A/B testing, monitoring, and debugging (Pinaya et al., 2019).

(4) ML classifiers must also be stable in the sense that the results do not change substantially when the training data is modified. Classification research requires stability since unstable classifier predictions might lead to difficulty repeating findings (Uddin et al., 2017; Itani and Thanou, 2021). We should also consider model reproducibility. Several proposed strategies, such as the Brain Imaging Data Structure (BIDS) (Rojas et al., 2006; Gorgolewski et al., 2016), try to standardize data structure, description, and storage.

(5) Previous studies lacked adequate neuroimaging training data. Using data-augmentation techniques to produce synthetic data from the given training dataset is an effective way to supplement data (Hussain et al., 2017; Shin et al., 2018). Flipping, cropping, translating, adding Gaussian noise, and blurring are data augmentation techniques (Shorten and Khoshgoftaar, 2019). Few-shot and zero-shot transfer learning techniques (Koch et al., 2015; Wu et al., 2020; Chen D. et al., 2020) can also help. No study exploits this type of learning to diagnose autism. In contrast, it has been applied to other diseases such as Alzheimer’s (Cheng et al., 2017).

(6) Training on datasets from diverse sources may be necessary to generalize better. It may be a promising method for learning adaptive classifiers (Chen et al., 2021) or applying to multitask learning that treats each site as a single task (Huang et al., 2020) in future studies to reduce the impact of dataset variability.

(7) There is an urgent need to gather and evaluate data according to ASD subgroups to better diagnosis and individualized therapy. We also advise focusing investigations on geographical datasets as ASD prevalence is geographically dependent (LeCun et al., 2010).

(8) Financial constraints stifle diagnostic innovation. Although sMRI is less expensive than other MRI modalities, it is still costly and unlikely to be used frequently outside densely populated areas or big institutions (Tate et al., 2020). Other neuroimaging methods (e.g., electroencephalography or near-infrared spectroscopy) are more clinically applicable and should be noted in the future.

(9) Furthermore, further research using different and complementary features is needed to investigate them. Effective and correct integration of different imaging data is an increasing challenge when acquiring data from different collection sites. However, it supports the idea that incorporating them may achieve optimal accuracy and show formal investigation (Mellema et al., 2019). In addition, the integration model must identify useful features in the classification.

(10) Finally, it should be noted that most high-performance computing techniques and ML algorithms are developed for 2D images, however, MRI are 3D or 4D data. Expanding ML architecture from 2D to 3D/4D increases parameters and runtime, restricting progress in recognizing psychological indicators. Big data and demands to interchange data from various sources will necessitate new approaches to speed up the ML journey. Of these scalable solutions are parallel algorithms that consider the CPU-GPU or CPU-Accelerator architecture. Parallel processing of MRI data and DL networks involved with ASD diagnosis are necessary components of future high-performance computing techniques. Although rarely used to date, a few HPC approaches have been proposed to analyze MRI data (Lusher et al., 2018; Eslami and Saeed, 2018).

## Conclusion

ASD presents many challenges for clinicians and researchers seeking to understand its biological basis and target it with drugs and interventions. Recent research has indicated that ML/DL algorithms and structural brain MRI data may diagnose ASD. While these studies are promising, they have not achieved the expected success in early and accurate diagnosis. ASD’s intricacy and disparities across clinical and research groups demand further work to develop diagnostic tools. More practical feature extraction and selection techniques, more data, and reliable, and interpretable ML or DL models are needed. Nevertheless, the results of the studies are useful in identifying dysfunctional brain regions and bring us closer to understanding the biological basis of this predominant disorder. While many critical issues remain to be addressed in the future, we anticipate these technologies will improve, provide better, more personalized diagnoses, and be available to clinicians soon.

## Author contributions

RB made a substantial contribution to the work by researching the literature, writing the manuscript, and modeling, explaining, and interpreting the results. AB read and synthesized sMRI knowledge specific to ASD classification. HB and SJ contributed to a critical review of the draft. The article has been finally approved by all authors for publication, and accountability for any part of the article is taken by all authors.

## Footnotes

1
https://esperr.github.io/pubmed-by-year/

## References

[B1] Abbasi B. Goldenholz D. M. (2019). Machine learning applications in epilepsy. *Epilepsia* 60 2037–2047. 10.1111/epi.16333m 31478577 PMC9897263

[B2] Ahmad H. A. Yu H. J. Miller C. G. (2014). “Medical imaging modalities,” in *Medical imaging in clinical trials* (Berlin: Springer), 3–26. 10.1007/978-1-84882-710-3_1

[B3] Akhavan Aghdam M. Sharifi A. Pedram M. M. (2018). Combination of rs-fmri and smri data to discriminate autism spectrum disorders in young children using deep belief network. *J. Digit. Imaging* 31 895–903. 10.1007/s10278-018-0093-8 29736781 PMC6261184

[B4] Ali M. T. Elnakieb Y. A. Shalaby A. Mahmoud A. Switala A. Ghazal M. (2021). “Autism classification using smri: A recursive features selection based on sampling from multi-level high dimensional spaces,” in *Proceedings of the 2021 IEEE 18th International Symposium on Biomedical Imaging (ISBI)* (Piscataway, NJ: IEEE), 267–270. 10.1109/ISBI48211.2021.9433973

[B5] Ali M. T. ElNakieb Y. Elnakib A. Shalaby A. Mahmoud A. Ghazal M. (2022). The role of structure MRI in diagnosing autism. *Diagnostics* 12:165. 10.3390/diagnostics12010165 35054330 PMC8774643

[B6] American Psychiatric Association (2013). *Diagnostic and statistical manual of mental disorders: Dsm-5.* Arlington, TX: American Psychiatric Association. 10.1176/appi.books.9780890425596

[B7] Arbabshirani M. R. Plis S. Sui J. Calhoun V. D. (2017). Single subject prediction of brain disorders in neuroimaging: Promises and pitfalls. *Neuroimage* 145 137–165. 10.1016/j.neuroimage.2016.02.079 27012503 PMC5031516

[B8] Bilgen I. Guvercin G. Rekik I. (2020). Machine learning methods for brain network classification: Application to autism diagnosis using cortical morphological networks. *J. Neurosci. Methods.* 343:108799. 10.1016/j.jneumeth.2020.108799 32574639

[B9] Chaddad A. Desrosiers C. Hassan L. Tanougast C. (2017). Hippocampus and amygdala radiomic biomarkers for the study of autism spectrum disorder. *BMC Neurosci.* 18:52. 10.1186/s12868-017-0373-0 28821235 PMC6389224

[B10] Chen T. Chen Y. Yuan M. Gerstein M. Li T. Liang H. (2020). The development of a practical artificial intelligence tool for diagnosing and evaluating autism spectrum disorder: Multicenter study. *JMIR Med. Inform.* 8:e15767. 10.2196/15767 32041690 PMC7244998

[B11] Chen D. Zhang L. Ma C. (2020). “A multimodal diagnosis predictive model of alzheimer’s disease with few-shot learning,” in *Proceedings of the 2020 International Conference on Public Health and Data Science (ICPHDS)* (Piscataway, NJ: IEEE), 273–277. 10.1109/ICPHDS51617.2020.00060

[B12] Chen R. Jiao Y. Herskovits E. H. (2011). Structural mri in autism spectrum disorder. *Pediatr. Res.* 69 63–68. 10.1203/PDR.0b013e318212c2b3 21289538 PMC3081653

[B13] Chen Y. Yan J. Jiang M. Zhao Z. Zhao W. Zhang R. (2021). “Attention-based node-edge graph convolutional networks for identification of autism spectrum disorder using multi-modal mri data,” in *Proceedings of the Chinese Conference on Pattern Recognition and Computer Vision (PRCV)* (Berlin: Springer), 374–385. 10.1007/978-3-030-88010-1_31

[B14] Cheng B. Liu M. Shen D. Li Z. Zhang D. (2017). Multi-domain transfer learning for early diagnosis of alzheimer’s disease. *Neuroinformatics* 15 115–132. 10.1007/s12021-016-9318-5 27928657 PMC5444948

[B15] Cortes C. Vapnik V. (1995). Support-vector networks. *Mach. Learn.* 20 273–297. 10.1007/BF00994018

[B16] Dai Y. Shi F. Wang L. Wu G. Shen D. (2013). ibeat: A toolbox for infant brain magnetic resonance image processing. *Neuroinformatics* 11 211–225. 10.1007/s12021-012-9164-z 23055044

[B17] de Belen R. A. J. Bednarz T. Sowmya A. Del Favero D. (2020). Computer vision in autism spectrum disorder research: A systematic review of published studies from 2009 to 2019. *Transl. Psychiatry* 10 1–20. 10.1038/s41398-020-01015-w 32999273 PMC7528087

[B18] Dekhil O. Ali M. El-Nakieb Y. Shalaby A. Soliman A. Switala A. (2021). A personalized autism diagnosis cad system using a fusion of structural mri and resting-state functional mri data. *Front. Psychiatry* 10:392. 10.3389/fpsyt.2019.00392 31333507 PMC6620533

[B19] Dekhil O. Ali M. Haweel R. Elnakib Y. Ghazal M. Hajjdiab H. (2020). “A comprehensive framework for differentiating autism spectrum disorder from neurotypicals by fusing structural MRI and resting state functional MRI,” in *Seminars in pediatric neurology*, Vol. 34 (Philadelphia, PA: WB Saunders), 100805. 10.1016/j.spen.2020.100805 32446442

[B20] Demirhan A. (2018). The effect of feature selection on multivariate pattern analysis of structural brain mr images. *Phys. Med.* 47 103–111. 10.1016/j.ejmp.2018.03.002 29609811

[B21] Devika K. Oruganti V. R. M. (2020). “Early classification of abnormal health using longitudinal structural mri data,” in *Proceedings of the 2020 IEEE 17th India Council International Conference (INDICON)* (Piscataway, NJ: IEEE), 1–6.

[B22] Di Martino A. O’connor D. Chen B. Alaerts K. Anderson J. S. Assaf M. (2017). Enhancing studies of the connectome in autism using the autism brain imaging data exchange ii. *Sci. Data* 4 1–15. 10.1038/sdata.2017.10 28291247 PMC5349246

[B23] Di Martino A. Yan C.-G. Li Q. Denio E. Castellanos F. X. Alaerts K. (2014). The autism brain imaging data exchange: Towards a large-scale evaluation of the intrinsic brain architecture in autism. *Mol. Psychiatry* 19 659–667. 10.1038/mp.2013.78 23774715 PMC4162310

[B24] Dicuonzo G. Galeone G. Shini M. Massari A. (2022). “Towards the use of big data in healthcare: A literature review,” in *Healthcare*, Vol. 10 (Basel: Multidisciplinary Digital Publishing Institute), 1232. 10.3390/healthcare10071232 35885759 PMC9322051

[B25] Du Y. Fu Z. Calhoun V. D. (2018). Classification and prediction of brain disorders using functional connectivity: Promising but challenging. *Front. Neurosci.* 12:525. 10.3389/fnins.2018.00525 30127711 PMC6088208

[B26] Dua M. Yadav R. Mamgai D. Brodiya S. (2020). An improved rnn-lstm based novel approach for sheet music generation. *Procedia Comput. Sci.* 171 465–474. 10.1016/j.procs.2020.04.049

[B27] Dukart J. Sambataro F. Bertolino A. Alzheimer’s Disease Neuroimaging Initiative (2016). Accurate prediction of conversion to Alzheimer’s disease using imaging, genetic, and neuropsychological biomarkers. *J. Alzheimers Dis.* 49 1143–1159. 10.3233/JAD-150570 26599054

[B28] Ecker C. Marquand A. Mourao-Miranda J. Johnston P. Daly E. M. Brammer M. J. (2010). ^∼^Describing the brain in autism in five dimensions—magnetic resonance imaging-assisted diagnosis of autism spectrum disorder using a multiparameter classification approach. *J. Neurosci.* 30 10612–10623. 10.1523/JNEUROSCI.5413-09.2010 20702694 PMC6634684

[B29] Eill A. Jahedi A. Gao Y. Kohli J. S. Fong C. H. Solders S. (2019). Functional connectivities are more informative than anatomical variables in diagnostic classification of autism. *Brain Connect.* 9 604–612. 10.1089/brain.2019.0689 31328535 PMC6798803

[B30] El Naqa I. Murphy M. J. (2022). “What are machine and deep learning?,” in *Machine and deep learning in oncology, medical physics and radiology* (Berlin: Springer), 3–15. 10.1007/978-3-030-83047-2_1

[B31] Eslami T. Almuqhim F. Raiker J. S. Saeed F. (2021). Machine learning methods for diagnosing autism spectrum disorder and attention-deficit/hyperactivity disorder using functional and structural mri: A survey. *Front. Neuroinform.* 14:62. 10.3389/fninf.2020.575999 33551784 PMC7855595

[B32] Eslami T. Saeed F. (2018). Fast-gpu-pcc: A gpu-based technique to compute pairwise pearson’s correlation coefficients for time series data—fmri study. *High Throughput* 7:11. 10.3390/ht7020011 29677161 PMC6023306

[B33] Eslami T. Mirjalili V. Fong A. Laird A. R. Saeed F. (2019). Asd-diagnet: A hybrid learning approach for detection of autism spectrum disorder using fmri data. *Front. Neuroinform.* 13:70. 10.3389/fninf.2019.00070 31827430 PMC6890833

[B34] Ferrari E. Bosco P. Calderoni S. Oliva P. Palumbo L. Spera G. (2020). Dealing with confounders and outliers in classification medical studies: The autism spectrum disorders case study. *Artif. Intell. Med.* 108:101926. 10.1016/j.artmed.2020.101926 32972657

[B35] Fischl B. (2012). Freesurfer. *Neuroimage* 62 774–781. 10.1016/j.neuroimage.2012.01.021 22248573 PMC3685476

[B36] Fu Y. Zhang J. Li Y. Shi J. Zou Y. Guo H. (2021). A novel pipeline leveraging surface-based features of small subcortical structures to classify individuals with autism spectrum disorder. *Prog. Neuro Psychopharmacol. Biol. Psychiatry* 104:109989. 10.1016/j.pnpbp.2020.109989 32512131 PMC9632410

[B37] Gao K. Sun Y. Niu S. Wang L. (2021). Unified framework for early-stage status prediction of autism based on infant structural magnetic resonance imaging. *Autism Res.* 14 2512–2523. 10.1002/aur.2626 34643325 PMC8665129

[B38] Gao K. Fan Z. Su J. Zeng L.-L. Shen H. Zhu J. (2022). Deep transfer learning for cerebral cortex using area-preserving geometry mapping. *Cereb. Cortex.* 32, 2972–2984. 34791082 10.1093/cercor/bhab394

[B39] Gao J. Chen M. Li Y. Gao Y. Li Y. Cai S. (2021). Multisite autism spectrum disorder classification using convolutional neural network classifier and individual morphological brain networks. *Front. Neurosci.* 14:1473. 10.3389/fnins.2020.629630 33584183 PMC7877487

[B40] Gargaro B. A. Rinehart N. J. Bradshaw J. L. Tonge B. J. Sheppard D. M. (2011). Autism and adhd: How far have we come in the comorbidity debate? *Neurosci. Biobehav. Rev.* 35 1081–1088. 10.1016/j.neubiorev.2010.11.002 21093480

[B41] Ghiassian S. Greiner R. Jin P. Brown M. R. (2016). Using functional or structural magnetic resonance images and personal characteristic data to identify adhd and autism. *PLoS One* 11:e0166934. 10.1371/journal.pone.0166934 28030565 PMC5193362

[B42] Gorgolewski K. J. Auer T. Calhoun V. D. Craddock R. C. Das S. Duff E. P. (2016). The brain imaging data structure, a format for organizing and describing outputs of neuroimaging experiments. *Sci. Data* 3 1–9. 10.1038/sdata.2016.44 27326542 PMC4978148

[B43] Gorriz J. M. Ramírez J. Segovia F. Martínez F. J. Lai M.-C. Lombardo M. V. (2019). A machine learning approach to reveal the neurophenotypes of autisms. *Int. J. Neural Syst.* 29:1850058. 10.1142/S0129065718500582 30782022

[B44] Graa O. Rekik I. (2019). Multi-view learning-based data proliferator for boosting classification using highly imbalanced classes. *J. Neurosci. Methods* 327:108344. 10.1016/j.jneumeth.2019.108344 31421161

[B45] Grimm O. Pohlack S. Cacciaglia R. Winkelmann T. Plichta M. M. Demirakca T. (2015). Amygdalar and hippocampal volume: A comparison between manual segmentation, freesurfer and vbm. *J. Neurosci. Methods* 253 254–261. 10.1016/j.jneumeth.2015.05.024 26057114

[B46] Guo W. Li H. Zhu Y. Lan L. Yang S. Drukker K. (2015). Prediction of clinical phenotypes in invasive breast carcinomas from the integration of radiomics and genomics data. *J. Med. Imaging* 2:041007. 10.1117/1.JMI.2.4.041007 26835491 PMC4718467

[B47] Guo X. Wang J. Wang X. Liu W. Yu H. Xu L. (2021). Diagnosing autism spectrum disorder in children using conventional mri and apparent diffusion coefficient based deep learning algorithms. *Eur. Radiol.* 32 761–770. 10.1007/s00330-021-08239-4 34482428

[B48] Hall D. Huerta M. F. McAuliffe M. J. Farber G. K. (2012). Sharing heterogeneous data: The national database for autism research. *Neuroinformatics* 10 331–339. 10.1007/s12021-012-9151-4 22622767 PMC4219200

[B49] Hashimoto T. Sasaki M. Fukumizu M. Hanaoka S. Sugai K. Matsuda H. (2000). Single-photon emission computed tomography of the brain in autism: Effect of the developmental level. *Pediatr. Neurol.* 23 416–420. 10.1016/S0887-8994(00)00224-1 11118797

[B50] Hazlett H. C. Gu H. McKinstry R. C. Shaw D. W. Botteron K. N. Dager S. R. (2012). Brain volume findings in 6-month-old infants at high familial risk for autism. *Am. J. Psychiatry* 169 601–608. 10.1176/appi.ajp.2012.11091425 22684595 PMC3744332

[B51] Hazlett H. C. Gu H. Munsell B. C. Kim S. H. Styner M. Wolff J. J. (2017). Early brain development in infants at high risk for autism spectrum disorder. *Nature* 542 348–351. 10.1038/nature21369 28202961 PMC5336143

[B52] Heinsfeld A. S. Franco A. R. Craddock R. C. Buchweitz A. Meneguzzi F. (2018). Identification of autism spectrum disorder using deep learning and the abide dataset. *Neuroimage Clin.* 17 16–23. 10.1016/j.nicl.2017.08.017 29034163 PMC5635344

[B53] Howell B. R. Styner M. A. Gao W. Yap P.-T. Wang L. Baluyot K. (2019). The unc/umn baby connectome project (bcp): An overview of the study design and protocol development. *Neuroimage* 185 891–905. 10.1016/j.neuroimage.2018.03.049 29578031 PMC6151176

[B54] Huang Z.-A. Liu R. Tan K. C. (2020). “Multi-task learning for efficient diagnosis of asd and adhd using resting-state fmri data,” in *Proceedings of the 2020 International Joint Conference on Neural Networks (IJCNN)* (Piscataway: IEEE), 1–7. 10.1109/IJCNN48605.2020.9206852

[B55] Hussain Z. Gimenez F. Yi D. Rubin D. (2017). “Differential data augmentation techniques for medical imaging classification tasks,” in *Proceedings of the AMIA Annual Symposium*, Vol. 2017, (Bethesda: American Medical Informatics Association), 979. PMC5977656 29854165

[B56] Ibrahim S. Djemal R. Alsuwailem A. (2018). Electroencephalography (eeg) signal processing for epilepsy and autism spectrum disorder diagnosis. *Biocybern. Biomed. Eng.* 38 16–26. 10.1016/j.bbe.2017.08.006

[B57] Irimia A. Lei X. Torgerson C. M. Jacokes Z. J. Abe S. Van Horn J. D. (2018). Support vector machines, multidimensional scaling and magnetic resonance imaging reveal structural brain abnormalities associated with the interaction between autism spectrum disorder and sex. *Front. Comput. Neurosci.* 12:93. 10.3389/fncom.2018.00093 30534065 PMC6276724

[B58] Islam J. (2019). *Towards AI-assisted disease diagnosis: Learning deep feature representations for medical image analysis.* Ph.D. thesis. Atlanta: Georgia State University.

[B59] Itani S. Thanou D. (2021). Combining anatomical and functional networks for neuropathology identification: A case study on autism spectrum disorder. *Med. Image Anal.* 69:101986. 10.1016/j.media.2021.101986 33610918

[B60] Jarraya S. K. Masmoudi M. Hammami M. (2021). A comparative study of autistic children emotion recognition based on spatio-temporal and deep analysis of facial expressions features during a meltdown crisis. *Multimed. Tools Appl.* 80 83–125. 10.1007/s11042-020-09451-y

[B61] Jenkinson M. Beckmann C. F. Behrens T. E. Woolrich M. W. Smith S. M. (2012). Fsl. *Neuroimage* 62 782–790. 10.1016/j.neuroimage.2011.09.015 21979382

[B62] Kanner L. (1943). Autistic disturbances of affective contact. *Nerv. Child* 2 217–250. 4880460

[B63] Ke F. Yang R. (2020). Classification and biomarker exploration of autism spectrum disorders based on recurrent attention model. *IEEE Access* 8 216298–216307. 10.1109/ACCESS.2020.3038479 27534393

[B64] Ke F. Choi S. Kang Y. H. Cheon K.-A. Lee S. W. (2020). Exploring the structural and strategic bases of autism spectrum disorders with deep learning. *IEEE Access* 8 153341–153352. 10.1109/ACCESS.2020.3016734

[B65] Khodatars M. Shoeibi A. Sadeghi D. Ghaasemi N. Jafari M. Moridian P. (2021). Deep learning for neuroimaging-based diagnosis and rehabilitation of autism spectrum disorder: A review. *Comput. Biol. Med.* 139:104949. 10.1016/j.compbiomed.2021.104949 34737139

[B66] Kijonka M. Borys D. Psiuk-Maksymowicz K. Gorczewski K. Wojcieszek P. Kossowski B. (2020). Whole brain and cranial size adjustments in volumetric brain analyses of sex-and age-related trends. *Front. Neurosci.* 14:278. 10.3389/fnins.2020.00278 32317915 PMC7147247

[B67] Kim Y.-K. Na K.-S. (2018). Application of machine learning classification for structural brain mri in mood disorders: Critical review from a clinical perspective. *Prog. Neuro Psychopharmacol. Biol. Psychiatry* 80 71–80. 10.1016/j.pnpbp.2017.06.024 28648568

[B68] Koch G. Zemel R. Salakhutdinov R. (2015). *Siamese neural networks for one-shot image recognition.* Lille: ICML deep learning workshop.

[B69] Kong Y. Gao J. Xu Y. Pan Y. Wang J. Liu J. (2019). Classification of autism spectrum disorder by combining brain connectivity and deep neural network classifier. *Neurocomputing* 324 63–68. 10.1016/j.neucom.2018.04.080

[B70] Krizhevsky A. Sutskever I. Hinton G. E. (2012). Imagenet classification with deep convolutional neural networks. *Adv. Neural Inf. Process. Syst.* 25 1097–1105.

[B71] Kumar R. Pandey A. K. Baz A. Alhakami H. Alhakami W. Agrawal A. (2020). Fuzzy-based symmetrical multi-criteria decision-making procedure for evaluating the impact of harmful factors of healthcare information security. *Symmetry* 12:664. 10.3390/sym12040664

[B72] Landhuis E. (2020). Deep learning takes on tumours. *Nature* 580 551–554. 10.1038/d41586-020-01128-8 32317799

[B73] LeCun Y. Kavukcuoglu K. Farabet C. (2010). “Convolutional networks and applications in vision,” in *Proceedings of the 2010 IEEE International Symposium on Circuits and Systems* (Piscataway: IEEE), 253–256. 10.1109/ISCAS.2010.5537907

[B74] Lee J. D. Meadan H. (2021). Children with autism spectrum disorders in low-resource settings: Reported experiences and needs of parents in mongolia. *J. Autism Dev. Disord.* 51 3586–3599. 10.1007/s10803-020-04818-4 33387240

[B75] Leming M. J. Baron-Cohen S. Suckling J. (2021). Single-participant structural similarity matrices lead to greater accuracy in classification of participants than function in autism in mri. *Mol. Autism* 12 1–15. 10.1186/s13229-021-00439-5 33971956 PMC8112019

[B76] Li G. Chen M.-H. Li G. Wu D. Lian C. Sun Q. (2019a). “A longitudinal mri study of amygdala and hippocampal subfields for infants with risk of autism,” in *Proceedings of the International Workshop on Graph Learning in Medical Imaging* (Berlin: Springer), 164–171. 10.1007/978-3-030-35817-4_20 PMC7043018 32104792

[B77] Li G. Chen M.-H. Li G. Wu D. Sun Q. Shen D. (2019b). “A preliminary volumetric mri study of amygdala and hippocampal subfields in autism during infancy,” in *Proceedings of the 2019 IEEE 16th International Symposium on Biomedical Imaging (ISBI 2019)* (Piscataway: IEEE), 1052–1056. 10.1109/ISBI.2019.8759439 PMC6824593 31681457

[B78] Li X. Dvornek N. C. Papademetris X. Zhuang J. Staib L. H. Ventola P. (2018). “2-channel convolutional 3d deep neural network (2cc3d) for fmri analysis: Asd classification and feature learning,” in *Proceedings of the 2018 IEEE 15th International Symposium on Biomedical Imaging (ISBI 2018)* (Piscataway: IEEE), 1252–1255. 10.1109/ISBI.2018.8363798 PMC7519578 32983370

[B79] Li H. Parikh N. A. He L. (2018). A novel transfer learning approach to enhance deep neural network classification of brain functional connectomes. *Front. Neurosci.* 12:491. 10.3389/fnins.2018.00491 30087587 PMC6066582

[B80] Li G. Liu M. Sun Q. Shen D. Wang L. (2018). “Early diagnosis of autism disease by multi-channel cnns,” in *Proceedings of the International Workshop on Machine Learning in Medical Imaging* (Berlin: Springer), 303–309. 10.1007/978-3-030-00919-9_35 PMC6235442 30450494

[B81] Libero L. E. DeRamus T. P. Lahti A. C. Deshpande G. Kana R. K. (2015). Multimodal neuroimaging-based classification of autism spectrum disorder using anatomical, neurochemical, and white matter correlates. *Cortex* 66 46–59. 10.1016/j.cortex.2015.02.008 25797658 PMC4782775

[B82] Liu G.-D. Li Y.-C. Zhang W. Zhang L. (2020). A brief review of artificial intelligence applications and algorithms for psychiatric disorders. *Engineering* 6 462–467. 10.1016/j.eng.2019.06.008 34174282

[B83] Lord C. Bishop S. Anderson D. (2015). Developmental trajectories as autism phenotypes. *Am. J. Med. Genet. Part C Semin. Med. Genet.* 169 198–208. 10.1002/ajmg.c.31440 25959391 PMC4898819

[B84] Lusher J. Ji J. Orr J. (2018). High-performance correlation and mapping engine for rapid generating brain connectivity networks from big fmri data. *J. Comput. Sci.* 26 157–164. 10.1016/j.jocs.2018.04.013 30440567

[B85] Manzanera O. M. Meles S. K. Leenders K. L. Renken R. J. Pagani M. Arnaldi D. (2019). Scaled subprofile modeling and convolutional neural networks for the identification of parkinson’s disease in 3d nuclear imaging data. *Int. J. Neural Syst.* 29:1950010. 10.1142/S0129065719500102 31046514

[B86] Mellema C. Treacher A. Nguyen K. Montillo A. (2019). “Multiple deep learning architectures achieve superior performance diagnosing autism spectrum disorder using features previously extracted from structural and functional mri,” in *Proceedings of the 2019 IEEE 16th International Symposium on Biomedical Imaging (ISBI 2019)* (Piscataway: IEEE), 1891–1895. 10.1109/ISBI.2019.8759193 PMC6859452 31741704

[B87] Mishra M. Pati U. C. (2021). “Autism spectrum disorder detection using surface morphometric feature of smri in machine learning,” in *Proceedings of the 2021 8th International Conference on Smart Computing and Communications (ICSCC)* (Piscataway: IEEE), 17–20. 10.1109/ICSCC51209.2021.9528240

[B88] Misman M. F. Samah A. A. Ezudin F. A. Majid H. A. Shah Z. A. Hashim H. (2019). “Classification of adults with autism spectrum disorder using deep neural network,” in *Proceedings of the 2019 1st International Conference on Artificial Intelligence and Data Sciences (AiDAS)* (Piscataway: IEEE), 29–34. 10.1109/AiDAS47888.2019.8970823

[B89] Mittal S. Umesh S. (2021). A survey on hardware accelerators and optimization techniques for rnns. *J. Syst. Arch.* 112:101839. 10.1016/j.sysarc.2020.101839

[B90] Moradi E. Khundrakpam B. Lewis J. D. Evans A. C. Tohka J. (2017). Predicting symptom severity in autism spectrum disorder based on cortical thickness measures in agglomerative data. *Neuroimage* 144 128–141. 10.1016/j.neuroimage.2016.09.049 27664827

[B91] Morris C. Rekik I. (2017). “Autism spectrum disorder diagnosis using sparse graph embedding of morphological brain networks,” in *Graphs in Biomedical Image Analysis, Computational Anatomy and Imaging Genetics* (Berlin: Springer), 12–20. 10.1007/978-3-319-67675-3_2

[B92] Mostapha M. (2020). *Learning from complex neuroimaging datasets.* Ph.D. thesis. Chapel Hill, NC: The University of North Carolina at Chapel Hill.

[B93] Nogay H. S. Adeli H. (2020). Machine learning (ml) for the diagnosis of autism spectrum disorder (asd) using brain imaging. *Rev. Neurosci.* 31 825–841. 10.1515/revneuro-2020-0043 32866134

[B94] Nomi J. S. Uddin L. Q. (2015). Developmental changes in large-scale network connectivity in autism. *Neuroimage Clin.* 7 732–741. 10.1016/j.nicl.2015.02.024 25844325 PMC4375789

[B95] Ozonoff S. Young G. S. Carter A. Messinger D. Yirmiya N. Zwaigenbaum L. (2011). Recurrence risk for autism spectrum disorders: A baby siblings research consortium study. *Pediatrics* 128 e488–e495. 10.1542/peds.2010-2825 21844053 PMC3164092

[B96] Pagnozzi A. M. Conti E. Calderoni S. Fripp J. Rose S. E. (2018). A systematic review of structural mri biomarkers in autism spectrum disorder: A machine learning perspective. *Int. J. Dev. Neurosci.* 71 68–82. 10.1016/j.ijdevneu.2018.08.010 30172895

[B97] Panja S. Chatterjee A. Yasmin G. (2018). “Kernel functions of svm: A comparison and optimal solution,” in *Proceedings of the International Conference on Advanced Informatics for Computing Research* (Berlin: Springer), 88–97. 10.1007/978-981-13-3140-4_9

[B98] Parisot S. Ktena S. I. Ferrante E. Lee M. Guerrero R. Glocker B. (2018). Disease prediction using graph convolutional networks: Application to autism spectrum disorder and Alzheimer’s disease. *Med. Image Anal.* 48 117–130. 10.1016/j.media.2018.06.001 29890408

[B99] Payakachat N. Tilford J. M. Ungar W. J. (2016). National database for autism research (ndar): Big data opportunities for health services research and health technology assessment. *Pharmacoeconomics* 34 127–138. 10.1007/s40273-015-0331-6 26446859 PMC4761298

[B100] Peng L. Lin L. Lin Y. Chen Y.-W. Mo Z. Vlasova R. M. (2021). Longitudinal prediction of infant mr images with multi-contrast perceptual adversarial learning. *Front. Neurosci.* 15:653213. 10.3389/fnins.2021.653213 34566556 PMC8458966

[B101] Phan A. C. Phan T. C. Trieu T. N. (2022). A systematic approach to healthcare knowledge management systems in the era of big data and artificial intelligence. *Appl. Sci.* 12:4455. 10.3390/app12094455

[B102] Pinaya W. H. Mechelli A. Sato J. R. (2019). Using deep autoencoders to identify abnormal brain structural patterns in neuropsychiatric disorders: A large-scale multi-sample study. *Hum. Brain Mapp.* 40 944–954. 10.1002/hbm.24423 30311316 PMC6492107

[B103] Polsek D. Jagatic T. Cepanec M. Hof P. Simić G. (2011). Recent developments in neuropathology of autism spectrum disorders. *Transl. Neurosci.* 2 256–264. 10.2478/s13380-011-0024-3 22180840 PMC3237008

[B104] Quaak M. van de Mortel L. Thomas R. M. van Wingen G. (2021). Deep learning applications for the classification of psychiatric disorders using neuroimaging data: Systematic review and meta-analysis. *Neuroimage Clin.* 30:102584. 10.1016/j.nicl.2021.102584 33677240 PMC8209481

[B105] Raamana P. R. Strother S. C. (2020). Does size matter? the relationship between predictive power of single-subject morphometric networks to spatial scale and edge weight. *Brain Struct. Funct.* 225 2475–2493. 10.1007/s00429-020-02136-0 32945910

[B106] Rakic’ M. Cabezas M. Kushibar K. Oliver A. Llado X. (2020). Improving the detection of autism spectrum disorder by combining structural and functional mri information. *Neuroimage Clin.* 25:102181. 10.1016/j.nicl.2020.102181 31982680 PMC6994708

[B107] Richards R. Greimel E. Kliemann D. Koerte I. K. Schulte-Korne G. Reuter M. (2020). Increased hippocampal shape asymmetry and volumetric ventricular asymmetry in autism spectrum disorder. *Neuroimage Clin.* 26:102207. 10.1016/j.nicl.2020.102207 32092683 PMC7037573

[B108] Rojas D. C. Peterson E. Winterrowd E. Reite M. L. Rogers S. J. Tregellas J. R. (2006). Regional gray matter volumetric changes in autism associated with social and repetitive behavior symptoms. *BMC Psychiatry* 6:56. 10.1186/1471-244X-6-56 17166273 PMC1770914

[B109] Rojas-Domínguez A. Padierna L. C. Valadez J. M. C. Puga-Soberanes H. J. Fraire H. J. (2017). Optimal hyper-parameter tuning of SVM classifiers with application to medical diagnosis. *IEEE Access* 6 7164–7176. 10.1109/ACCESS.2017.2779794

[B110] Saeys Y. Inza I. Larranaga P. (2007). A review of feature selection techniques in bioinformatics. *Bioinformatics* 23 2507–2517. 10.1093/bioinformatics/btm344 17720704

[B111] Samuel A. L. (2000). Some studies in machine learning using the game of checkers. *IBM J. Res. Dev.* 44 206–226. 10.1147/rd.441.0206 33813791

[B112] Sejdic E. Falk T. H. (Eds.) (2018). *Signal processing and machine learning for biomedical big data.* Boca Raton: CRC Press. 10.1201/9781351061223

[B113] Sen B. Borle N. C. Greiner R. Brown M. R. (2018). A general prediction model for the detection of adhd and autism using structural and functional mri. *PLoS One* 13:e0194856. 10.1371/journal.pone.0194856 29664902 PMC5903601

[B114] Seyedi S. Jafari R. Talaei A. Naseri S. Momennezhad M. Moghaddam M. D. (2020). Comparing vbm and roi analyses for detection of gray matter abnormalities in patients with bipolar disorder using mri. *Middle East Curr. Psychiatry* 27 1–7. 10.1186/s43045-020-00076-3

[B115] Shahamat H. Abadeh M. S. (2020). Brain mri analysis using a deep learning based evolutionary approach. *Neural Netw.* 126 218–234. 10.1016/j.neunet.2020.03.017 32259762

[B116] Shen M. D. Kim S. H. McKinstry R. C. Gu H. Hazlett H. C. Nordahl C. W. (2017). Increased extra-axial cerebrospinal fluid in high-risk infants who later develop autism. *Biol. Psychiatry* 82 186–193. 10.1016/j.biopsych.2017.02.1095 28392081 PMC5531051

[B117] Shin H.-C. Tenenholtz N. A. Rogers J. K. Schwarz C. G. Senjem M. L. Gunter J. L. (2018). “Medical image synthesis for data augmentation and anonymization using generative adversarial networks,” in *Proceedings of the International Workshop on Simulation and Synthesis in Medical Imaging* (Berlin: Springer), 1–11. 10.1007/978-3-030-00536-8_1

[B118] Shorten C. Khoshgoftaar T. M. (2019). A survey on image data augmentation for deep learning. *J. Big Data* 6 1–48. 10.1186/s40537-019-0197-0 PMC8287113 34306963

[B119] Sivapalan S. Aitchison K. J. (2014). Neurological structure variations in individuals with autism spectrum disorder: A review. *Klinik Psikofarmakoloji Bulteni Bull. Clin. Psychopharmacol.* 24 268–275. 10.5455/bcp.20140903110206

[B120] Soussia M. Rekik I. (2018). Unsupervised manifold learning using high-order morphological brain networks derived from t1-w mri for autism diagnosis. *Front. Neuroinform.* 12:70. 10.3389/fninf.2018.00070 30459585 PMC6232924

[B121] Squarcina L. Nosari G. Marin R. Castellani U. Bellani M. Bonivento C. (2021). Automatic classification of autism spectrum disorder in children using cortical thickness and support vector machine. *Brain Behav.* 11:e2238. 10.1002/brb3.2238 34264004 PMC8413814

[B122] Suzuki K. (2013). Machine learning in computer-aided diagnosis of the thorax and colon in CT: A survey. *IEICE Trans. Inf. Syst.* 96 772–783. 10.1587/transinf.E96.D.772 24174708 PMC3810349

[B123] Tanu Kakkar D. (2019). Diagnostic assessment techniques and non-invasive biomarkers for autism spectrum disorder. *Int. J. E Health Med. Commun. (IJEHMC)* 10 79–95. 10.4018/IJEHMC.2019070105

[B124] Tate A. E. McCabe R. C. Larsson H. Lundstrom S. Lichtenstein P. Kuja-Halkola R. (2020). ^..^Predicting mental health problems in adolescence using machine learning techniques. *PLoS One* 15:e0230389. 10.1371/journal.pone.0230389 32251439 PMC7135284

[B125] Toro R. Traut N. Beggatio A. Heuer K. Varoquaux G. (2018). *IMPAC: Imaging-psychiatry challenge: Predicting autism a data challenge on autism spectrum disorder detection*.

[B126] Tummala S. (2021). “Deep learning framework using siamese neural network for diagnosis of autism from brain magnetic resonance imaging,” in *Proceedings of the 2021 6th International Conference for Convergence in Technology (I2CT)* (Piscataway: IEEE), 1–5. 10.1109/I2CT51068.2021.9418143

[B127] Uddin L. Q. Dajani D. Voorhies W. Bednarz H. Kana R. (2017). Progress and roadblocks in the search for brain-based biomarkers of autism and attention-deficit/hyperactivity disorder. *Transl. Psychiatry* 7 e1218–e1218. 10.1038/tp.2017.164 28892073 PMC5611731

[B128] Wang L. Li G. Shi F. Cao X. Lian C. Nie D. (2018). “Volume-based analysis of 6-month-old infant brain mri for autism biomarker identification and early diagnosis,” in *Proceedings of the International Conference on Medical Image Computing and Computer-Assisted Intervention* (Berlin: Springer), 411–419. 10.1007/978-3-030-00931-1_47 PMC6231401 30430147

[B129] Wang Z. Peng D. Shang Y. Gao J. (2021). Autistic spectrum disorder detection and structural biomarker identification using self-attention model and individual-level morphological covariance brain networks. *Front. Neurosci.* 15:756868. 10.3389/fnins.2021.756868 34712116 PMC8547518

[B130] World Health Organization (2013). *Meeting report: Autism spectrum disorders and other developmental disorders: From raising awareness to building capacity: World health organization, Geneva, Switzerland 16-18 september 2013.* Geneva: World Health Organization.

[B131] Wu J. Zhao Z. Sun C. Yan R. Chen X. (2020). Few-shot transfer learning for intelligent fault diagnosis of machine. *Measurement* 166:108202. 10.1016/j.measurement.2020.108202

[B132] Wujek B. Hall P. Günes F. (2016). *Best practices for machine learning applications.* Cary: SAS Institute Inc.

[B133] Xiao X. Fang H. Wu J. Xiao C. Xiao T. Qian L. (2017). Diagnostic model generated by mri-derived brain features in toddlers with autism spectrum disorder. *Autism Res.* 10 620–630. 10.1002/aur.1711 27874271

[B134] Xu M. Calhoun V. Jiang R. Yan W. Sui J. (2021). Brain imaging-based machine learning in autism spectrum disorder: Methods and applications. *J. Neurosci. Methods* 361:109271. 10.1016/j.jneumeth.2021.109271 34174282 PMC9006225

[B135] Yalçin A. Rekik I. (2021). A diagnostic unified classification model for classifying multi-sized and multi-modal brain graphs using graph alignment. *J. Neurosci. Methods* 348:109014. 10.1016/j.jneumeth.2020.109014 33309587

[B136] Yang G. Ye Q. Xia J. (2022). Unbox the black-box for the medical explainable ai via multi-modal and multi-centre data fusion: A mini-review, two showcases and beyond. *Inf. Fusion* 77 29–52. 10.1016/j.inffus.2021.07.016 34980946 PMC8459787

[B137] Yassin W. Nakatani H. Zhu Y. Kojima M. Owada K. Kuwabara H. (2020). Machine learning classification using neuroimaging data in schizophrenia, autism, ultra-high risk and first-episode psychosis. *Transl. Psychiatry* 10 1–11. 10.1038/s41398-020-00965-5 32801298 PMC7429957

[B138] Yasuhara A. (2010). Correlation between eeg abnormalities and symptoms of autism spectrum disorder (asd). *Brain Dev.* 32 791–798. 10.1016/j.braindev.2010.08.010 20826075

[B139] Yin T. Ma P. Tian Z. Xie K. He Z. Sun R. (2020). Machine learning in neuroimaging: A new approach to understand acupuncture for neuroplasticity. *Neural Plast.* 2020:8871712. 10.1155/2020/8871712 32908491 PMC7463415

[B140] Zhang L. Wang M. Liu M. Zhang D. (2020). A survey on deep learning for neuroimaging-based brain disorder analysis. *Front. Neurosci.* 14:779. 10.3389/fnins.2020.00779 33117114 PMC7578242

[B141] Zhang M. Zhao X. Zhang W. Chaddad A. Evans A. Poline J. B. (2020). “Deep discriminative learning for autism spectrum disorder classification,” in *Proceedings of the International Conference on Database and Expert Systems Applications* (Berlin: Springer), 435–443. 10.1007/978-3-030-59003-1_29

[B142] Zhang W. Wang Y. (2020). “Deep multimodal brain network learning for joint analysis of structural morphometry and functional connectivity,” in *Proceedings of the 2020 IEEE 17th International Symposium on Biomedical Imaging (ISBI)* (Piscataway: IEEE), 1–5. 10.1109/ISBI45749.2020.9098624 PMC8130829 34012504

[B143] Zhang Z. Li G. Xu Y. Tang X. (2021). Application of artificial intelligence in the mri classification task of human brain neurological and psychiatric diseases: A scoping review. *Diagnostics* 11:1402. 10.3390/diagnostics11081402 34441336 PMC8392727

[B144] Zheng W. Eilam-Stock T. Wu T. Spagna A. Chen C. Hu B. (2019). Multi-feature-based network revealing the structural abnormalities in autism spectrum disordermoradi2017predicting. *IEEE Trans. Affect. Comput.* 12 732–742. 10.1109/TAFFC.2018.2890597

[B145] Zollei L. Iglesias J. E. Ou Y. Grant P. E. Fischl B. (2020). Infant freesurfer: An automated ^..^segmentation and surface extraction pipeline for t1-weighted neuroimaging data of infants 0–2 years. *Neuroimage* 218:116946. 10.1016/j.neuroimage.2020.116946 32442637 PMC7415702

